# AI-generated language remains systematically distinguishable from human communication

**DOI:** 10.1073/pnas.2531748123

**Published:** 2026-10-12

**Authors:** Nicolò Pagan, Petter Törnberg, Christopher A. Bail, Christopher Barrie

**Affiliations:** ^a^ Department of Informatics, University of Zurich, Zurich CH-8006, Switzerland; ^b^ Institute for Logic, Language and Computation, University of Amsterdam, Amsterdam 1098-xg, The Netherlands; ^c^ Department of Sociology, Computer Science, Political Science, and Public Policy, Duke University, Durham, NC 27708; ^d^ Department of Sociology, New York University, New York, NY 10003; ^e^ Department of Sociology, University of Oxford, Oxford OX1 1JD, United Kingdom

**Keywords:** large language models, AI agent validation, social simulation, AI detection, Human–AI communication

## Abstract

Large language models (LLMs) are increasingly used in the social sciences to simulate human behavior and communication, yet validation methods for these applications remain limited. Existing evaluations often rely on human judgments of plausibility, a coarse, difficult-to-scale, and often unreliable benchmark for distinguishing AI-generated from human text. As a result, researchers lack robust tools for assessing the realism of LLM-generated language and for calibrating models to real-world data. We address this gap in two ways. First, we introduce the Conversational Human Authenticity Test (CHAT), a validation framework that combines aggregate metrics of detectability and semantic similarity with interpretable linguistic features, including stylistic markers and topical patterns, to assess how closely LLMs approximate human language in a given dataset. Second, we benchmark nine open-weight LLMs across five calibration strategies, including fine-tuning, stylistic prompting, and context retrieval, on user interactions from X, Bluesky, and Reddit. We find that even after calibration, LLM outputs remain clearly distinguishable from human text, especially in affective tone and emotional expression. Instruction-tuned models generally underperform base models, and larger models do not obviously improve human-likeness. We also identify a trade-off between realism and semantic fidelity: methods that make outputs seem more human often move them further from the content of human responses. Together, these results provide a scalable framework for validation and calibration in LLM-based social simulation while underscoring current limits in reproducing human communication.

## Significance

Researchers in the social sciences are increasingly using large language models (LLMs) to simulate human behavior and communication. Yet robust, systematic methods for assessing how faithfully these models reproduce human expression remain limited. We introduce a framework for measuring how closely AI-generated text resembles human language and apply it to nine open-weight models across three social media platforms and multiple calibration strategies. We find that even the best-calibrated systems remain distinguishable from human users, particularly in emotional and affective expression. Larger models do not consistently improve human-likeness, and reducing detectability often lowers semantic fidelity to human responses. Together, these results identify important limits on the use of current LLMs as proxies for human communication in social science research.

Large language models (LLMs) have rapidly become indispensable tools across the social sciences, powering tasks from text annotation and survey design to synthetic data generation (1–3). Increasingly, researchers use LLMs not only as instruments of analysis but as proxies for human behavior. By simulating realistic dialog and decision-making processes, LLMs promise to act as consistent and scalable stand-ins for human participants (4, 5), opening new horizons for generative social simulations that go beyond traditional agent-based models (6–8). These applications rest on a crucial assumption: that LLMs can convincingly reproduce human communication.

Establishing this assumption requires addressing two fundamental challenges (9, 10): validation—determining whether model outputs are truly realistic—and calibration—aligning models with the linguistic and emotional patterns of human communication (11–13). Current approaches fall short on both fronts. Calibration largely relies on ad hoc prompt engineering rather than systematic analysis or postgeneration selection (14, 15). Consequently, it remains unclear which calibration strategies meaningfully improve human-likeness, and under what conditions, as most approaches are neither systematically compared nor evaluated against consistent benchmarks.

In the social simulation literature, validation still often relies on human judgment, asking whether text “sounds” human (6, 16). Studies typically rely on crowd-workers or expert evaluations to assess plausibility (17–22), yet such tests are subjective, unscalable, and set a low bar: humans routinely overlook subtle linguistic and emotional cues that distinguish authentic from synthetic text (9, 23). A parallel strand of work uses LLMs themselves as evaluators to assess realism and coherence (19, 24–26), despite evidence that such methods are poorly calibrated and exhibit self-favoring biases (27, 28). Taken together, existing approaches often conflate surface plausibility with the deeper question of whether LLMs faithfully reproduce the structure of human communication.

A related literature focuses on detecting AI-generated text. Although this work is not primarily concerned with validation for social simulation, it offers tools that are highly relevant to that problem, since any systematic differences that allow machine detection may also reveal where LLM-generated language departs from human communication. Studies in NLP and computational social science show that LLM-generated text exhibits distinctive stylistic patterns—often appearing longer, more polite, more articulate, and more structured than human communication (9, 29–31). Some studies use quantitative methods to assess similarity, including embedding-based cosine similarity (32), perplexity scores (33), keyword and content distributions (34), or stance alignment with real-world data (31). These findings align with a broader machine learning literature showing that AI-generated text can be reliably distinguished from human text using automated detection methods (35–37). However, this detection literature is not designed to validate LLMs as behavioral proxies. Its main objective is typically to identify synthetic content “in the wild,” often through black-box classifiers that provide limited insight into how and where AI-generated text diverges from human communication. For researchers seeking to use LLMs in social simulation, those divergences are the central issue. This leaves an important gap: we know that computational methods can detect differences between human and AI-generated text, but we lack interpretable validation frameworks specifically designed to assess whether LLMs can serve as faithful proxies for human participants.

We address both gaps. First, we develop what we term Conversational Human Authenticity Test (CHAT)—a systematic validation framework that adapts detection methods for the specific purpose of evaluating LLM-generated social media content—in effect a scalable, automated counterpart to the classic Turing test (38), replacing a human interrogator’s subjective judgment with reproducible, quantitative metrics. Our framework combines three complementary dimensions: 1) detectability, measuring how easily human and AI text can be distinguished using machine learning classifiers; 2) semantic fidelity, quantifying similarity in meaning to human reference replies; and 3) interpretable linguistic analysis, identifying the stylistic and topical features that reveal AI authorship. By integrating aggregate detection metrics with interpretable diagnostics, CHAT reveals not only whether LLM-generated text differs from human text, but also how and where these differences emerge.

Second, we conduct a comprehensive calibration benchmark, evaluating how different calibration strategies affect human-likeness. We test nine open-weight LLMs under two levels of calibration. The first compares four prompt-engineering configurations—baseline, persona descriptions, stylistic examples, context retrieval—and fine-tuning. The second applies postgeneration selection, generating 20 candidate replies and selecting the best either for semantic similarity (cosine-optimal) or for minimal detectability (ML-optimal). This systematic comparison addresses the lack of standardized calibration practices in generative social simulation (9).

We use our validation framework to benchmark the models’ abilities to reproduce user interactions on X (formerly Twitter), Bluesky, and Reddit. Social media provides an important testbed: it is central to model training data and a key domain of applied social simulation (1, 33, 39–41).

Our findings indicate that even advanced models remain distinguishable from human text (c.f., 35, 36, 37). The validation framework identifies AI-generated text with 70 to 80% accuracy—well above chance. While calibration can mitigate some structural differences, deeper emotional and affective cues persist as reliable discriminators. Some sophisticated strategies, such as fine-tuning and persona descriptions, fail to improve realism or even make text more detectable, whereas simple stylistic examples and context retrieval yield modest gains.

Most notably, we identify a fundamental trade-off between realism and meaning: optimizing for reduced detectability lowers semantic fidelity, while optimizing for semantic fidelity does not consistently reduce detectability. While prior work has examined trade-offs in language generation (37), our finding concerns a specific tension between stylistic human-likeness (classifier-based detectability in social media) and semantic fidelity to actual human responses (similarity to ground-truth), with direct implications for simulation validity. Together, these findings show that stylistic human-likeness and semantic fidelity are competing rather than aligned objectives.

## Data, Models, and Metrics

### Data.

Our dataset comprises social media conversations collected from three platforms: Twitter/X (42), Bluesky (43), and Reddit (44). For each platform, we split users into training and held-out test sets and randomly sample 20 reply messages per test user; exact sample sizes and construction are reported in *Materials and Methods*.

### Models.

Our objective is to simulate user responses to social media messages and evaluate how closely AI-generated replies align with human responses in style, semantics, and tone. We therefore prompted LLMs to produce platform-appropriate replies to real social media messages while emulating each user’s linguistic style and conversational behavior.

We evaluated nine open-weight LLMs spanning six model families: Apertus, DeepSeek, Gemma, Llama, Mistral, and Qwen, focusing on compact 4–8B models alongside a larger Llama 3.1 70B for scale comparison; full model citations are given in *Materials and Methods*. For brevity, figures hereafter refer to these models by shortened labels: Apertus-8B, DeepSeek-R1-8B, Gemma-3-4B, Llama-8B, Llama-8B-Inst, Llama-70B, Mistral-7B, Mistral-7B-Inst, and Qwen-7B-Inst, respectively. Each model was run using standard sampling defaults for open-ended generation (temperature T=0.8, nucleus sampling P=0.9, top-k sampling k=50); *Materials and Methods* details this choice and reports a decoding hyperparameter ablation. Prompts included an LLM-generated persona description built from each user’s training-set messages, adapted for each platform (*Materials and Methods* and *SI Appendix*, sections 1 and 2).

### CHAT.

Our evaluation comprises both aggregate quality metrics and fine-grained interpretable analysis. We first assess overall generation quality through two complementary lenses: automated detectability and semantic preservation. We then conduct a deeper analysis to understand which specific linguistic characteristics distinguish AI-generated from human-authored content.

#### Detectability: BERT-based classification test.

We assess how effectively LLMs generate replies that remain indistinguishable from human-authored content when evaluated by an automated classifier. We pair each AI-generated reply with the human ground-truth reply to the same message and train a BERT-based classifier to discriminate between them, using only the reply text (not the parent message), so that classification reflects intrinsic linguistic style rather than topical or contextual differences. Higher classification accuracy indicates *more* distinguishable AI-generated content, with accuracy approaching 50% (random chance), suggesting successful human mimicry. Training and validation procedures are detailed in *Materials and Methods*.

#### Semantic matching: Cosine similarity score.

We examine the semantic similarity between AI-generated text and the corresponding human ground truth by embedding each reply into a dense vector space and computing the cosine similarity between AI-generated and human embeddings (embedding model detailed in *Materials and Methods*). This metric provides a complementary perspective to detectability, measuring how well AI-generated content preserves the semantic content of human responses.

#### Interpretable feature analysis: Random forest model and empath library.

While the above metrics provide aggregate measures of distinguishability and similarity, they do not reveal which specific linguistic characteristics drive these differences. We therefore employ two complementary approaches: a Random Forest classifier trained on interpretable lexical, stylistic, and structural features, and the Empath library (45), which quantifies topical and affective categories and identifies those that differ significantly between AI-generated and human-authored text. The full feature set and statistical procedures are listed in *Materials and Methods*.

## Benchmark Results for the Reference Configuration

We begin by using our validation framework to evaluate the established reference configuration for calibrating LLMs to generate human-like messages.

### BERT-Based Classification Test.

**Fig. 1.**
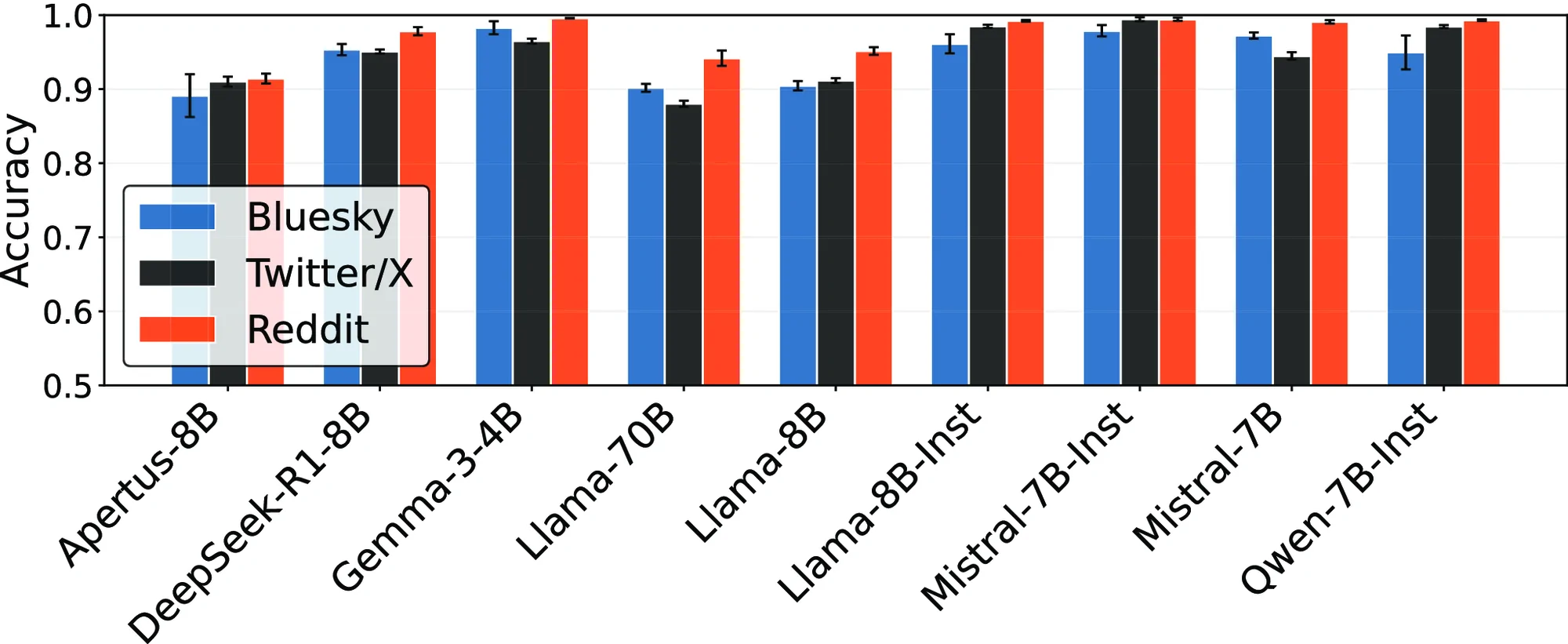
Results of the BERT-based classification test across datasets and models. Lower accuracy indicates better human mimicry (50% = chance level).

As shown in Fig. 1, the BERT classifier achieves consistently high accuracy across all models and datasets, never falling below 87%. This indicates that, within our benchmark, current state-of-the-art LLMs produce replies that remain readily distinguishable from human-authored content when evaluated by automated classifiers. Furthermore, we observe notable variation across model types, with non-instruction-tuned variants from Llama and Apertus achieving the lowest detection rates (i.e., best human mimicry). Platform-specific differences also emerge: the Twitter/X dataset tends to yield lower classification accuracy compared to Bluesky and Reddit, suggesting that most LLMs more successfully replicate the conversational patterns of Twitter/X users. Conversely, Reddit is consistently the most difficult to mimic. This may reflect either the distinctive stylistic characteristics of different platforms or their varying representation in LLM training corpora.

### Cosine Similarity.

**Fig. 2.**
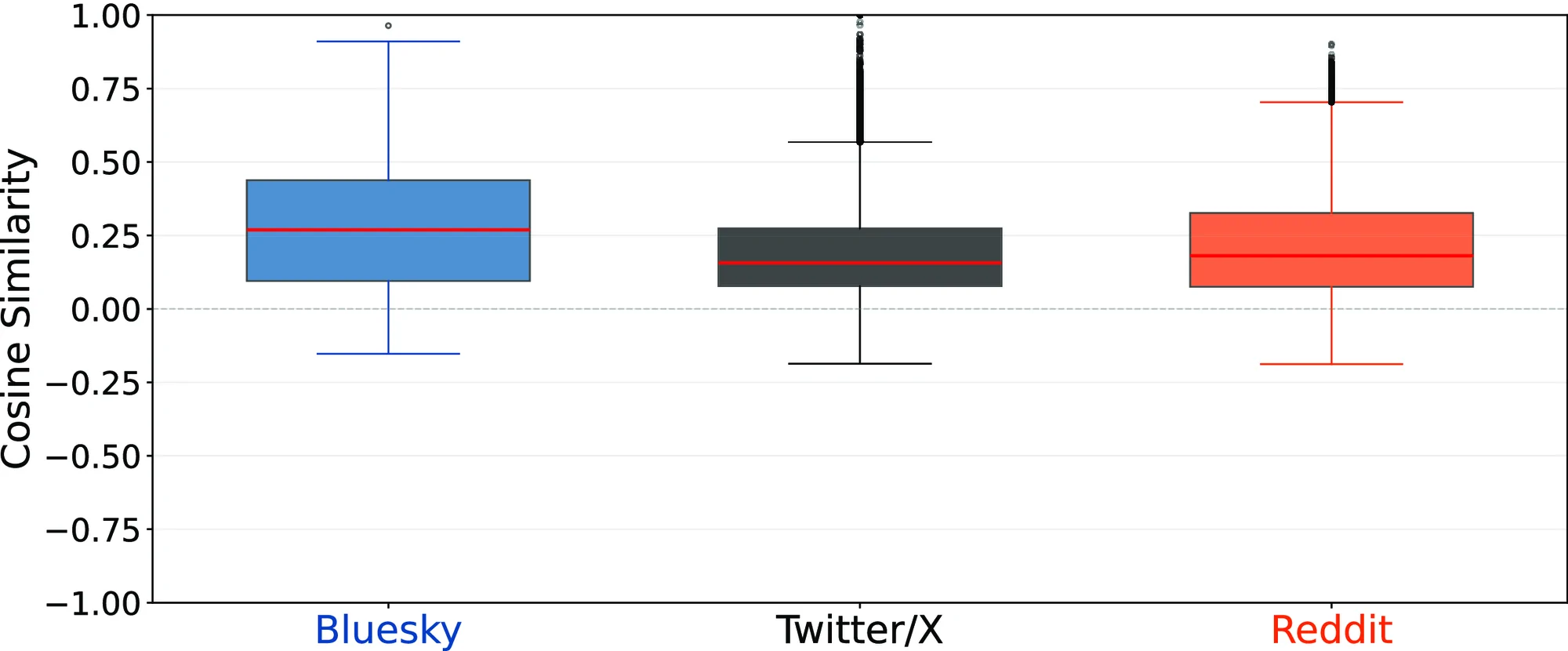
Distribution of content similarity between the AI-generated response and the corresponding human (ground-truth) response. Results are aggregated across different generating models. *SI Appendix*, Fig. S2 splits the results by model.

We next compare each AI-generated reply to the observed human reply using cosine similarity of sentence embeddings. Fig. 2 shows modest similarity between AI-generated and human-authored replies, with median scores ranging from about 0.2 for Twitter/X to 0.3 for Bluesky and with broadly similar patterns across LLMs.

These scores should be read as a reference point, not as a test of whether the model reproduced one uniquely correct human response. On social media, many different replies could plausibly fit the same post, but our data contain only one observed human reply for each message. Cosine similarity therefore tells us how close the model comes to that observed reply in meaning; it does not tell us whether the model produced the only valid response.

To help interpret these values, *SI Appendix*, section 8 reports several comparison benchmarks (*SI Appendix*, Fig. S6). Human–AI similarity is higher than the similarity between two replies written by the same person in different conversations, suggesting that generated replies capture more than a user’s general writing style, although this comparison does not hold the prompt constant. The clearer benchmark compares replies to the same source post. Under the reference configuration, replies generated for different simulated users are substantially more similar to one another than are replies written by two real humans responding to the same post, while repeated generations for the same simulated user are more similar still. This same-post comparison is well powered on Reddit (1,371 same-context pairs) but rests on only 18 pairs on Twitter/X, so we treat the Reddit estimate as reliable and the Twitter/X estimate as exploratory corroboration. On Reddit, for example, median AI–AI similarity is about 0.36, compared with a human–human baseline of about 0.19. This gap narrows as stylistic examples, retrieved context, and fine-tuning are added, and is nearly eliminated on Reddit after fine-tuning. However, deeper calibration does not consistently increase similarity to the response the target user actually produced: Adding style and context improves human–AI similarity on Reddit, but fine-tuning does not, and human–AI similarity declines across configurations on Twitter/X (exploratory, given the small same-post sample there).

Overall, these results suggest that AI-generated replies capture part of the meaning of human responses, but still differ substantially from the observed human continuation. Some of this gap likely reflects the difficulty of the task: the model must generate a reply from limited context, whereas the human author had access to the full conversational situation. Still, the consistency of this pattern across models suggests a broader limitation: current systems can produce semantically related replies without yet matching the range and variation of human responses. This motivates our next step, which examines the specific linguistic features that continue to distinguish AI-generated from human-authored text.

### Interpretability Results.

To understand which specific linguistic characteristics distinguish AI-generated from human-authored content, we examine both stylistic features through Random Forest classification and topical patterns through Empath analysis.

#### Stylistic and structural features.

**Fig. 3.**
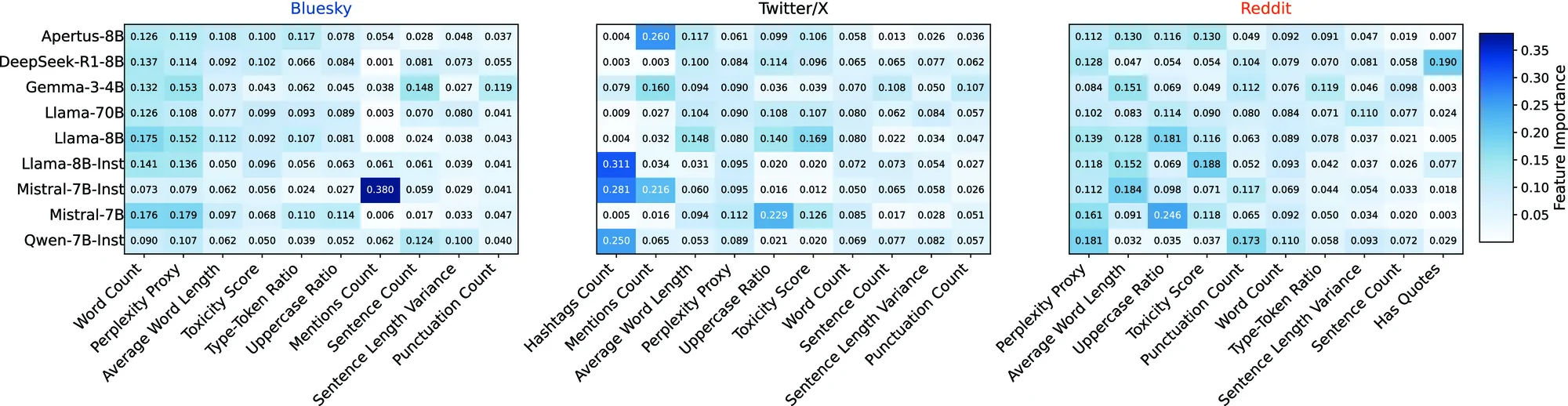
Feature importance (Mean Decrease in Impurity) from the Random Forest model classifying AI-generated vs. human text; top 10 features per dataset and model, ordered *Left* to *Right* by decreasing importance across models. Beyond platform/model-specific features (hashtags, mentions, uppercase ratio), word count, average word length, perplexity proxy, and toxicity score are consistently most important.

The Random Forest classifier reveals both platform-specific and model-specific patterns in feature importance (computed using Mean Decrease in Impurity, more details available in *SI Appendix*, section 6.A). As shown in Fig. 3, toxicity score consistently emerges as a critical discri minator across all three platforms, appearing among the top features for nearly all models. Beyond toxicity, platform-specific patterns emerge in the discriminative features. For Bluesky, word count, average word length, and perplexity proxy show relatively high importance across models, suggesting that AI-generated content differs in verbosity, as well as lexical sophistication and diversity. Twitter/X exhibits a distinct pattern: Hashtag count, mentions count, average word length, and perplexity proxy emerge as strong discriminators. The prominence of platform-specific features (hashtags, mentions) suggests that AI struggles to replicate the distinctive stylistic conventions of Twitter/X discourse. Reddit classification shows smaller variability across models but consistently relies on perplexity proxy, average word length, and uppercase ratio, pointing to differences in linguistic complexity and response elaboration.

Notably, the relative importance of specific features varies substantially across models within each platform, as evidenced by the varying shades across rows. Some models show pronounced reliance on particular features (darker cells), while others distribute importance more evenly. For instance, on Twitter/X, Llama-3.1-8B-Instruct, Mistral-7B-Instruct-v0.2, and Qwen-2.5-7B-Instruct show particularly strong signals from hashtag count. This heterogeneity suggests that different LLM architectures fail to replicate human writing in distinct ways, with each model producing distinctive linguistic signatures that enable classification. To complement these results, in *SI Appendix*, Fig. S3 we discuss model-level differences by analyzing the feature importance averaged across platforms, while in *SI Appendix*, Fig. S5 we report the direction and magnitude of AI-human differences for the top ten most discriminative features per model/platform. Key patterns include systematic underproduction of toxic content by instruction-tuned models, dramatic overuse of hashtags and mentions relative to near-zero human rates, and a verbosity mismatch in which most models substantially exceed human reply length.

#### Topical and affective patterns.

**Fig. 4.**
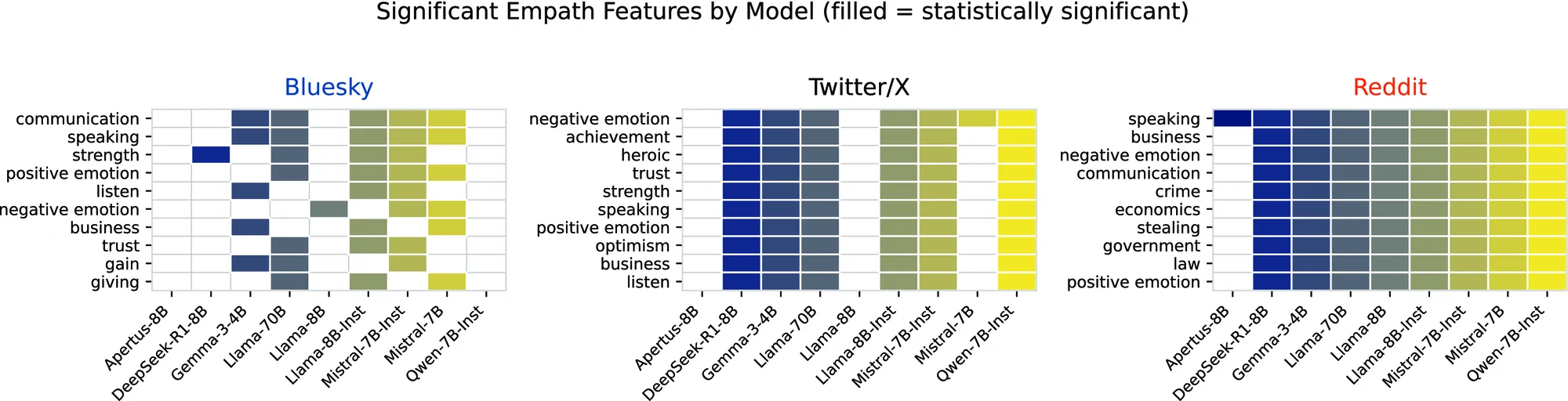
Empath topical and affective divergence for reference configurations (45): For each dataset, the top 10 Empath categories most frequently significant (Wilcoxon rank-sum, FDR-corrected *P* < 0.05) between AI-generated and human text, ordered *Top* to *Bottom* by frequency of divergence; each column is one of the nine models, and a filled cell marks a significant difference from human text. Categories near the top are filled for nearly all models (e.g., “speaking” on Reddit is significant for all nine), indicating widespread failure to replicate human affective-topical patterns; emotional and social-relational categories are consistently the most challenging.

The Empath analysis reveals systematic divergences in topical and emotional content across all platforms (Fig. 4). We visualize the top 10 most frequently divergent Empath categories per platform as a features-by-models grid: a filled cell indicates that a given model’s replies differ significantly from human text on that category, so the specific pattern of which models drive each divergence can be read directly off the figure.

All models exhibit substantial difficulties in replicating human topical patterns, though the severity varies by model and plat form. On Reddit, eight of nine models are significant on all or nearly all of the top 10 most frequently divergent features, with only Apertus-8B differing sharply (significant on just one); Twitter/X shows a similar pattern, with six of seven models significant on all ten. Bluesky is more heterogeneous, with two models significant on 8 of the 10 features and two others on only one. Notably, Llama-3.1-8B—despite ranking among the least detectable models by the BERT classifier—is significant on all ten of Reddit’s most divergent features, suggesting that evading classifier detection does not necessarily correspond to topical and affective alignment with human content.

Across platforms, affective and social categories emerge as consistent discriminators: positive emotion, negative emotion, speaking, and communication appear prominently in the top differentiating features for multiple datasets. Platform-specific patterns also emerge. Twitter/X shows strong differences in categories like optimism, trust, and giving, while Reddit diverges notably on business, government, and power. These patterns suggest that LLMs struggle to replicate the affective tone and social-relational language characteristic of human social media discourse, with the specific topical gaps varying according to platform norms and conversational culture.

Together, these interpretable analyses reveal that the detectability of AI-generated content stems from both topical-affective gaps (particularly in emotional expression and social language, as shown by Empath) and stylistic-structural differences (especially in toxicity, lexical characteristics, and platform-specific conventions, as shown by Random Forest). These patterns persist across different LLM architectures and platforms, though the specific discriminative features vary, suggesting persistent limitations in current models’ ability to capture the full range of human conversational behavior.

## Calibration Strategies

### From Zero-Shot Prompting to Fine-Tuning.

The evaluation of the reference configuration reveals persistent gaps between AI-generated and human-authored content across semantic, stylistic, and topical dimensions. To investigate whether these gaps can be mitigated through enhanced generation strategies, we systematically evaluate five configurations of increasing complexity. Each configuration builds incrementally on the previous one, incorporating additional contextual information and model adaptation techniques designed to improve the fidelity of AI-generated replies to human writing patterns.

We test the following configurations:

1. **Baseline (BL)**: A simple prompt requesting the model to generate a reply to a given social media message, without any user-specific information or stylistic guidance.
2. **Persona (PE)**: The prompt is augmented with a user persona description, which is generated by Llama-3.1-8B-Instruct based on a sample set of the user’s replies in the training set. This persona captures the user’s interests, communication style, and typical topics of discussion. *This configuration corresponds to the reference method evaluated in the previous section.*
3. **Persona+Stylistic Examples (PE+SE)**: Building on the persona description, the prompt includes 10 examples of the user’s prior replies drawn from the training set, providing concrete demonstrations of their writing style.
4. **Persona+Stylistic Examples+Context Retrieval (PE+SE+ CR)**: The prompt is further augmented with user-specific contextual information retrieved from prior posts, using a similarity-based retrieval method similar to the approach proposed in ref. 46. This provides relevant background information that may inform the reply.
5. **Persona+Stylistic Examples+Context Retrieval+Fine-tuning (PE+SE+CR+FT)**: The base model is fine-tuned on the same users being evaluated, in batches of up to 60 users, using the Parameter-Efficient Fine-Tuning (PEFT) library (47), adapting the model’s parameters to the user’s specific writing patterns (fine-tuning specifications in *Materials and Methods*). The fine-tuned model is then prompted with persona, examples, and retrieved context.

We apply these configurations across all nine LLMs and three datasets, evaluating their impact on the metrics established in our validation framework: BERT classification accuracy (detectability), cosine similarity (semantic fidelity), Random Forest feature importance (stylistic alignment), and Empath feature divergence (topical alignment). By comparing the reference (Persona) configuration against both simpler (Baseline) and more sophisticated approaches (PE+SE, PE+SE+CR, PE+SE+CR+FT), this systematic evaluation allows us to identify which calibration strategies most effectively reduce the gap between AI-generated and human-authored social media content, and whether different platforms or models benefit differentially from specific approaches.

### Accuracy Improvement in BERT-Based Classification Test.

**Fig. 5.**
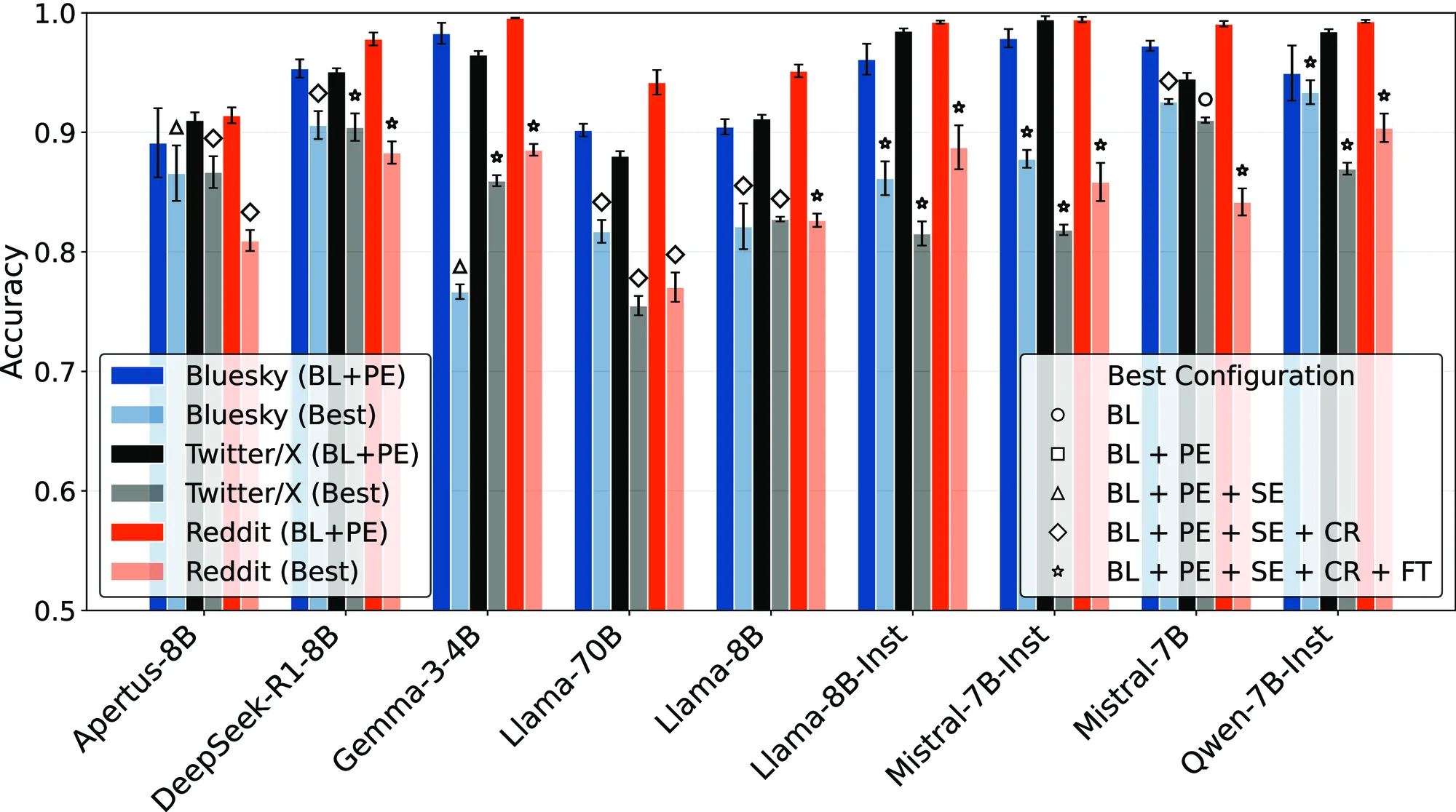
BERT classifier accuracy comparing the reference method (Baseline+Persona) and best-performing configurations across models and datasets. Increasing configuration complexity substantially reduces detectability and narrows the gap between models; the best configuration typically adds stylistic examples and context, or—especially for instruction-tuned models—fine-tuning.

Fig. 5 presents the BERT classification accuracy for the best-performing configuration of each model, compared against the reference configuration (Baseline+Persona) evaluated in the previous section. The results demonstrate that increasing configuration complexity substantially improves human-likeness: the best configurations achieve markedly lower classification accuracies than the reference configuration across all models and datasets, with improvements of roughly 10% and peaks reaching 15 to 20%.

This improvement is particularly pronounced for the previously low-performing instruction-tuned models, which benefit more from additional calibration steps, and in particular from fine-tuning. The absolute performance hierarchy observed in the reference configuration persists: non-instruction-tuned variants (Llama-3.1-8B, Llama-3.1-70B, and Apertus-8B-2509) almost consistently achieve the lowest classification accuracies (i.e., lowest detectability, with values ranging between 75% and 85%). Notably, calibration reduces the variance across models: the best configurations produce more uniform performance, with differences between models diminishing substantially compared to the reference configuration. Platform-specific patterns also remain consistent, with Twitter/X content generally exhibiting the lowest detectability.

**Fig. 6.**
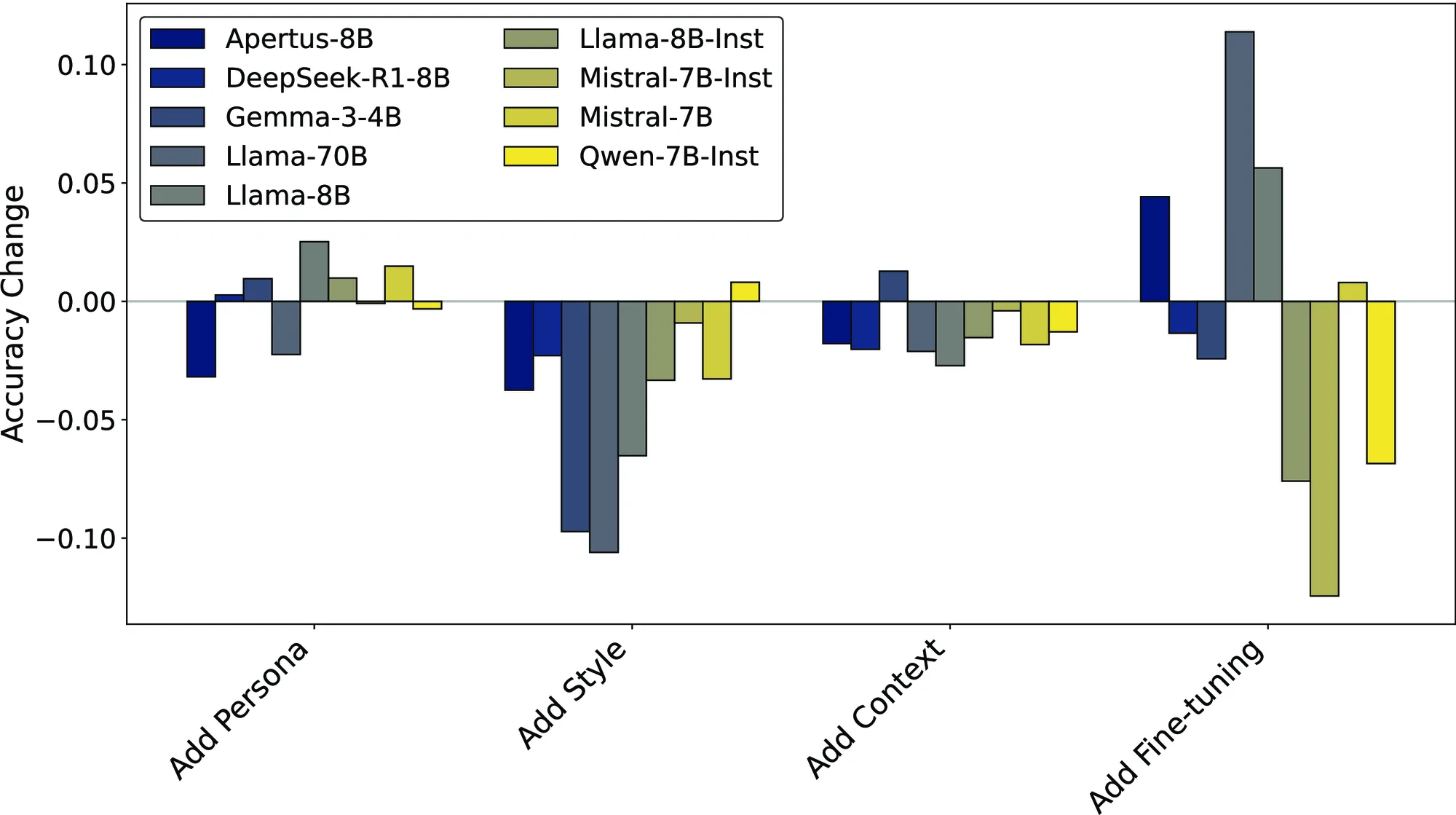
Stepwise impact of calibration strategies on BERT classifier accuracy, aggregated across datasets. Each point represents the change in accuracy when adding a new component: BL→PE (baseline to persona), PE→PE+SE (adding stylistic examples), PE+SE→PE+SE+CR (adding context retrieval), and PE+SE+CR→PE+SE+CR+FT (adding fine-tuning). Negative changes (downward) indicate reduced detectability and improved human mimicry.

A closer examination of the stepwise progression through configurations nonetheless reveals a surprising pattern. Fig. 6 illustrates how each incremental enhancement affects detection performance. Counterintuitively, not all calibration steps reduce detectability. The transition from baseline to persona (BL→PE) produces negligible or even slightly positive changes in accuracy for most models, indicating that adding abstract persona descriptions does not improve, and may even deteriorate, human-likeness. Similarly, fine-tuning (PE+SE+CR→PE+SE+CR+FT) shows highly variable effects, with some models experiencing substantial increases in detectability, particularly among non-instruction-tuned variants. An ablation study varying LoRA hyperparameters confirms that this counterproductive effect is robust to hyperparameter choice and is not attributable to overfitting (*SI Appendix*, section 11).

In stark contrast, two interventions consistently reduce detectability across nearly all models: adding stylistic examples (PE→PE+SE) and incorporating context retrieval (PE+SE→PE+SE+CR). These steps produce the most uniform improvements, with stylistic examples yielding classification accuracy reductions of approximately 2 to 10% and context retrieval contributing an additional 2 to 3% across models. The consistency of these effects suggests that concrete demonstrations of writing style and relevant contextual information address fundamental gaps in LLM-generated content that abstract descriptions or parameter adaptation cannot overcome.

These findings challenge conventional assumptions about what makes AI-generated text more human-like. While persona descriptions and fine-tuning might seem like sophisticated calibration strategies, they appear less effective, and sometimes counterproductive, compared to providing concrete stylistic examples and relevant context. This pattern suggests that current LLMs struggle less with adapting their general capabilities to specific users and more with accessing the specific stylistic and contextual cues that inform natural human responses.

### Semantic Similarity Declines Despite Reduced Detectability.

**Fig. 7.**
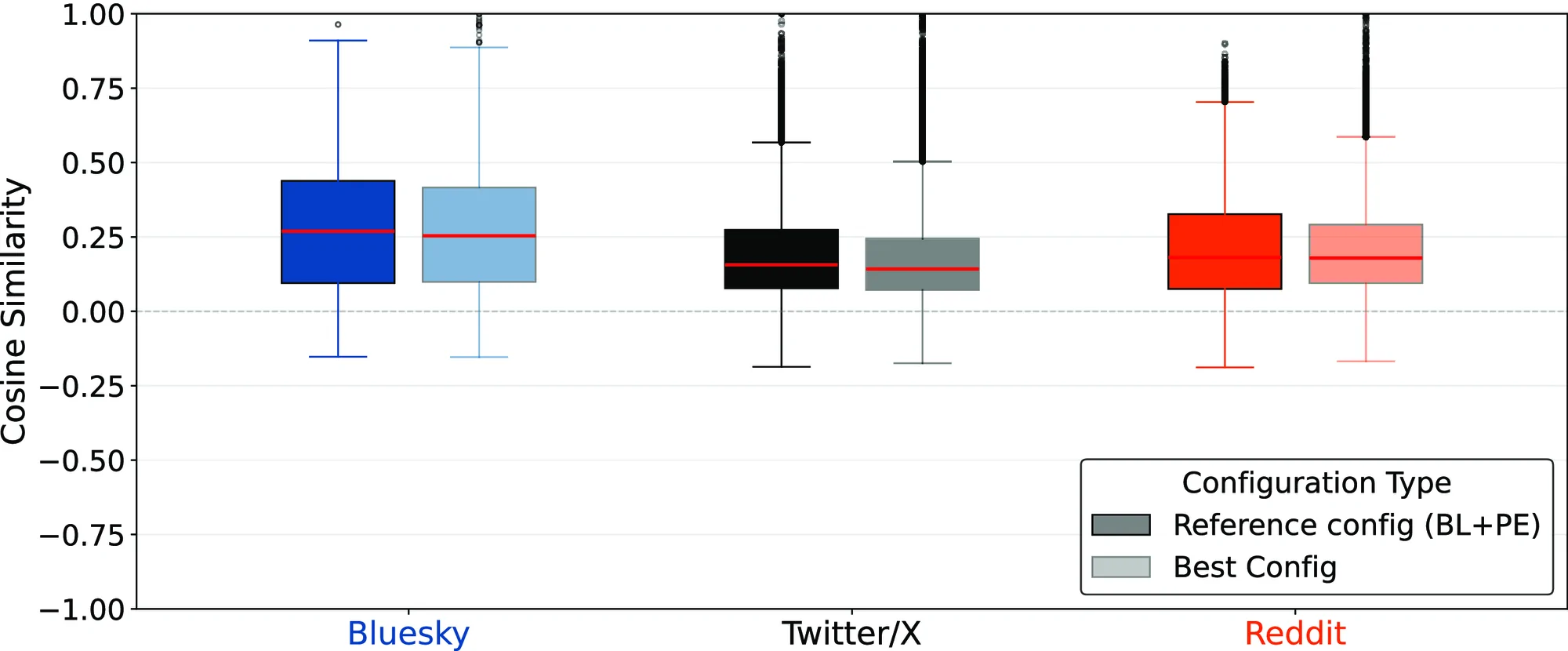
Distribution of cosine similarity scores between AI-generated and ground-truth human replies, comparing reference (BL+PE) and best-performing configurations across datasets. Median scores decline by 0.02 for Bluesky (0.27→0.25) and Twitter/X (0.16→0.14), and by 0.01 for Reddit (0.18→0.17). Box plots show median (red line), interquartile range (box), and full distribution excluding outliers (whiskers).

While the calibration strategies substantially reduce BERT classification accuracy, they also lead to decreased semantic similarity to ground-truth human replies. Fig. 7 compares the cosine similarity distributions between the reference configuration (Baseline+Persona) and the best-performing configurations identified in the previous section. Across all three platforms, the best configurations show slightly lower median cosine similarity scores than the reference configuration, revealing an unexpected trade-off.

This finding reveals a tension in AI-generated content: optimizing for human-likeness as judged by classifiers can come at the expense of semantic fidelity to what humans actually say. The best-performing configurations, which incorporate stylistic examples and contextual information, appear to prioritize matching the general stylistic and affective characteristics of human social media discourse over reproducing the specific semantic content of individual reference replies. In other words, these models learn to write in a more human-like manner but not necessarily to say the same things humans would say in response to specific prompts.

This dissociation suggests that detectability and semantic alignment represent different dimensions of generation quality. Configurations that successfully evade classifiers do so by capturing the distributional properties of human writing style, tone, and affect, but this stylistic mimicry may actually obscure or dilute the semantic signal from the original context. The decline in cosine similarity indicates that as models become better at “sounding human,” they may drift further from contextually appropriate responses, trading semantic relevance for stylistic authenticity.

### Feature-Level Changes Under Calibration.

To understand how different calibration strategies alter the linguistic characteristics that distinguish AI-generated from human-authored content, we apply the same interpretable analysis framework to the best-performing configurations.

**Fig. 8.**
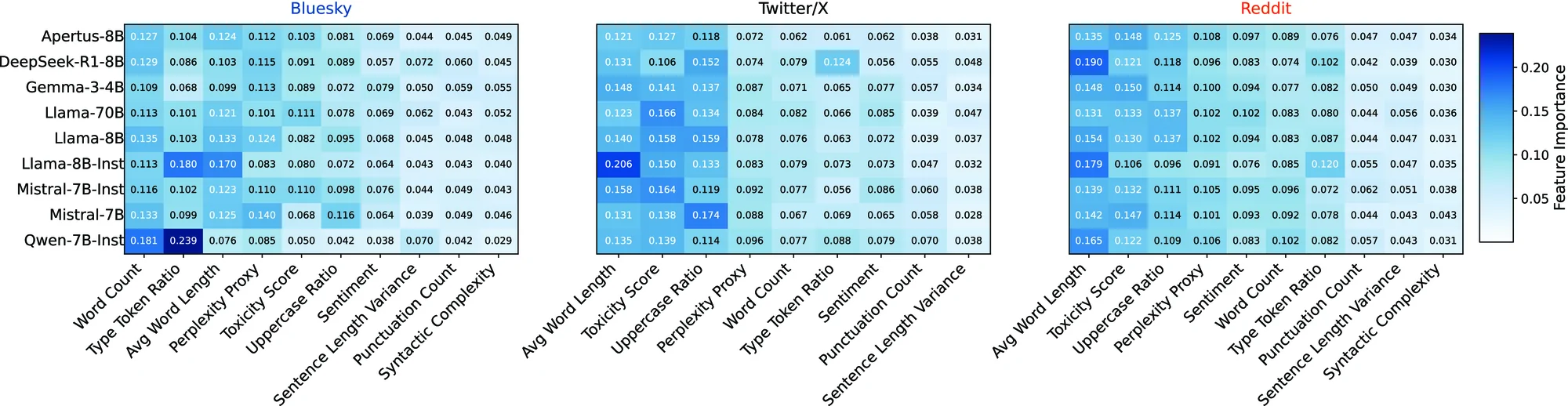
Random Forest feature importance for best-performing configurations (top 10 features per dataset/model, ordered by decreasing average importance; darker = more important). Compared to the reference configuration (Fig. 3), average word length becomes the dominant discriminator on two of three platforms and platform-specific formatting features (hashtags, mentions) diminish, while toxicity score persists—and even gains importance on Twitter/X and Reddit—indicating affective divergences remain despite calibration.

The Random Forest feature importance analysis (Fig. 8) reveals significant shifts in which features distinguish AI from human text compared to the reference configuration (Fig. 3). Most notably, average word length emerges as the dominant discriminator across two out of three platforms in optimized configurations, whereas it played a more modest role in the reference configuration. This suggests that calibration strategies, while reducing overall detectability, amplify (relatively to the other features) systematic differences in lexical sophistication.

Platform-specific patterns also shift substantially. In the optimized Bluesky configurations, the previous reliance on word count, perplexity proxy, average word length, and toxicity transforms into a focus on word count, type-token ratio, average word length, and perplexity proxy, with toxicity becoming less prominent. Twitter/X shows the most dramatic reorganization: the reference configuration emphasized hashtag count, mentions count, and average word length, while optimized configurations prioritize average word length, toxicity score, and uppercase ratio. This shift suggests that calibration successfully addresses platform-specific formatting conventions (hashtags, mentions) but places higher importance on linguistic complexity and affective intensity. Similarly, Reddit shows some reorganization on top, with perplexity proxy losing importance, but average word length and toxicity score gaining prominence in optimized configurations.

Critically, toxicity score persists as a discriminator across platforms, though its relative position varies. For Twitter/X and Reddit, toxicity actually increases in importance under calibration, appearing among the top three features. This indicates that calibration strategies do not address affective divergences as effectively as other features.

*SI Appendix*, section 9 mirrors this analysis at the feature-distribution level, showing raw AI vs. human distributions for the top discriminative features under each model’s optimal configuration (*SI Appendix*, Fig. S7). The dominant finding is that most stylometric biases documented under the reference configuration persist: toxicity suppression and verbosity mismatches remain largely unresolved, while mention overuse is substantially reduced. Sentiment emerges as a newly prominent discriminator under optimal configurations, suggesting that calibration introduces tonal variation that the classifier exploits.

**Fig. 9.**
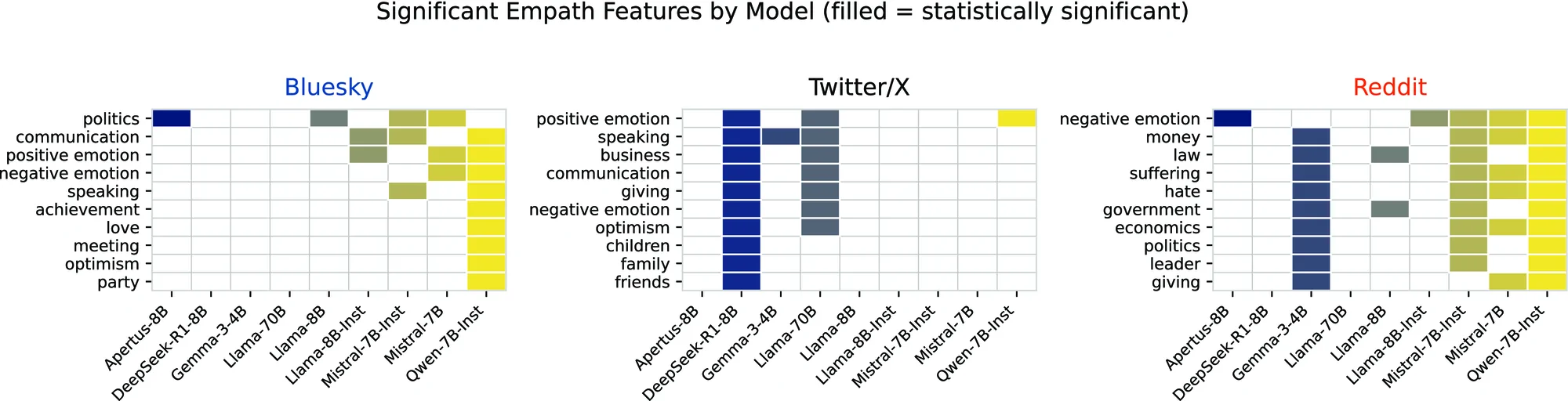
Empath topical and affective divergence for best-performing calibrated configurations (45), same grid as Fig. 4. Compared to the reference configuration, both the number of divergent features and the number of models responsible shrink substantially—most notably for Twitter/X, where only 2 models (DeepSeek-R1-8B, Llama-70B) show any significant divergence. Emotional categories (“positive emotion,” “negative emotion”) persist where differences remain, but the overall scope of divergence narrows substantially.

The Empath analysis reveals a striking reduction in topical-affective divergences under calibration, though certain emotional categories persist. Comparing Fig. 9 to the reference configuration (Fig. 4), the number of statistically significant differences decreases drastically across all platforms. For Twitter/X, the change is most dramatic: The reference configuration showed 10 frequently divergent features across 6 models, while the optimized configuration reduces this to mainly 7 features across only 2 models, namely DeepSeek, Llama-3.1-70B. Bluesky shows a substantial reduction in the number of models with statistically significant features, with only one model (Qwen-2.5-7B-Instruct) still displaying a large number of features, while Reddit maintains more divergent features but still shows meaningful improvement.

Despite this overall reduction, emotional categories persist among the top discriminators where differences remain: “positive emotion” continues to appear for Bluesky and Twitter/X, while “negative emotion” remains prominent for Reddit. However, the scope of divergence narrows considerably. Many affective and social categories that showed widespread differences in the reference configuration (“communication,” “speaking”) either disappear entirely or show differences for only 1 to 2 models in optimized configurations. This indicates that calibration strategies successfully address many topical dimensions while a core set of emotional categories remains challenging. The dramatic reduction in divergent features, particularly for Twitter/X, where some models achieve near-perfect topical alignment, demonstrates that prompt engineering (and sometimes fine-tuning) can substantially improve topical-affective resemblance, even if they cannot fully eliminate all emotional divergences.

### Postgeneration Selection.

While the previous sections focus on improving generation quality through prompt engineering and fine-tuning, we now investigate whether postgeneration selection from multiple candidates can further reduce detectability. Following best-of-N sampling approaches (48), we let each model produce 20 replies per test message. Among them, we then select an optimal candidate using two distinct selection procedures. This multicandidate approach offers key advantages: it enables exploration of natural variation in model outputs and allows us to identify which linguistic features can be mitigated through selection versus those that represent fundamental model limitations.

We define two selection procedures optimizing for different dimensions of human-likeness:

### Cosine Similarity Selection.

This procedure selects, for each prompt, the candidate reply with the highest cosine similarity (using the same embedding model as above) to the ground-truth human reply, directly optimizing for semantic alignment with the actual human response.

### ML-Optimal Selection.

The second procedure employs a machine learning classifier to identify the candidate reply most likely to be misclassified as human-authored, using the same interpretable feature set described in *Materials and Methods*. The set of LLM-generated replies is partitioned into folds by user, and for each fold we train a Random Forest classifier (100 estimators, fixed random seed) on the remaining folds to estimate each held-out candidate’s likelihood of being misclassified as human. The reply with the highest misclassification probability is selected.

The two postgeneration selection procedures capture complementary aspects of human-likeness: cosine similarity emphasizes semantic alignment with the actual human response, while the ML-optimal selection emphasizes stylistic and affective resemblance to human writing patterns in general. Because the cosine method relies directly on the reference reply while the ML-optimal selection does not, the two approaches are not strictly comparable but provide different perspectives on achievable improvements through postgeneration selection.

To understand the relationship between these selection methods and the variability in generated outputs, we examine the overlap between replies selected by each method. The overlap rate between ML-optimal and cosine similarity selection averages approximately 5 to 10% across models and datasets, barely exceeding the 5% random chance baseline (1 in 20 candidates). This minimal overlap confirms that the two methods optimize for fundamentally different characteristics and select distinct replies.

### Impact of Postgeneration Selection Strategies.

**Fig. 10.**
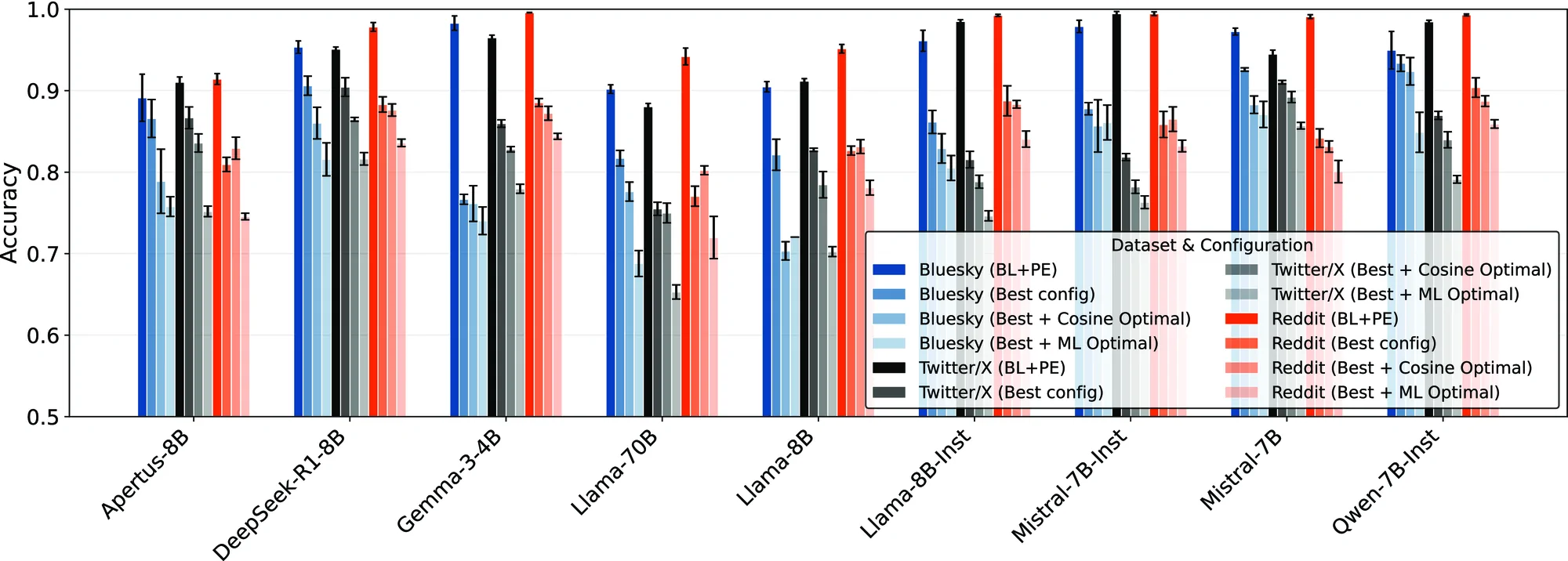
BERT classifier accuracy comparing four conditions per model: reference (BL+PE, darkest), best calibrated configuration, and that configuration with Cosine-Optimal (highest semantic similarity to the reference reply) or ML-Optimal (highest misclassification probability) selection. Error bars: SD across three seeds; lower = better mimicry.

Fig. 10 compares the effectiveness of different postgeneration selection strategies applied to the best-performing configurations. For each model and dataset, we evaluate four conditions: the reference configuration (BL+PE), the best prompt-optimized configuration identified previously (Best config), and two postgeneration selection methods applied to the best configuration (cosine similarity selection and ML-optimal selection).

Postgeneration selection yields modest but measurable improvements in reducing detectability. The ML-optimal selection consistently achieves the lowest BERT classification accuracies across most models and datasets, typically reducing accuracy by 5 to 10 percentage points compared to the best configuration without selection. Cosine similarity selection shows more variable performance: while it sometimes approaches ML-optimal effectiveness, it often performs comparably to or only marginally better than the best configuration alone. This asymmetry reflects the different postgeneration selection targets: ML-optimal selection directly optimizes for fooling a classifier with interpretable features correlated with BERT detectability, while cosine similarity optimizes for semantic alignment with the reference reply, which may not always correspond to reduced detectability.

Model-specific patterns reveal interesting variations. For some models (particularly Llama-3.1-8B, Llama-3.1-70B, and Apertus-8B-2509), ML-optimal selection produces substantial improvements, bringing accuracy closer to but never reaching the 50% chance level. For others, such as Qwen-2.5-7B-Instruct, the gains from postgeneration selection are often minimal, suggesting these models already generate relatively consistent outputs with limited exploitable variation across the 20 candidates.

Decomposing errors by class shows a consistent asymmetry across most models, platforms, and configurations (*SI Appendix*, section 13): The classifier is more likely to mistake a human reply for AI-generated text (a false positive) than to miss an actual AI-generated reply (a false negative), indicating that residual detectability reflects the overflagging of human writing at least as much as AI text failing to blend in.

#### Semantic similarity under postgeneration selection.

**Fig. 11.**
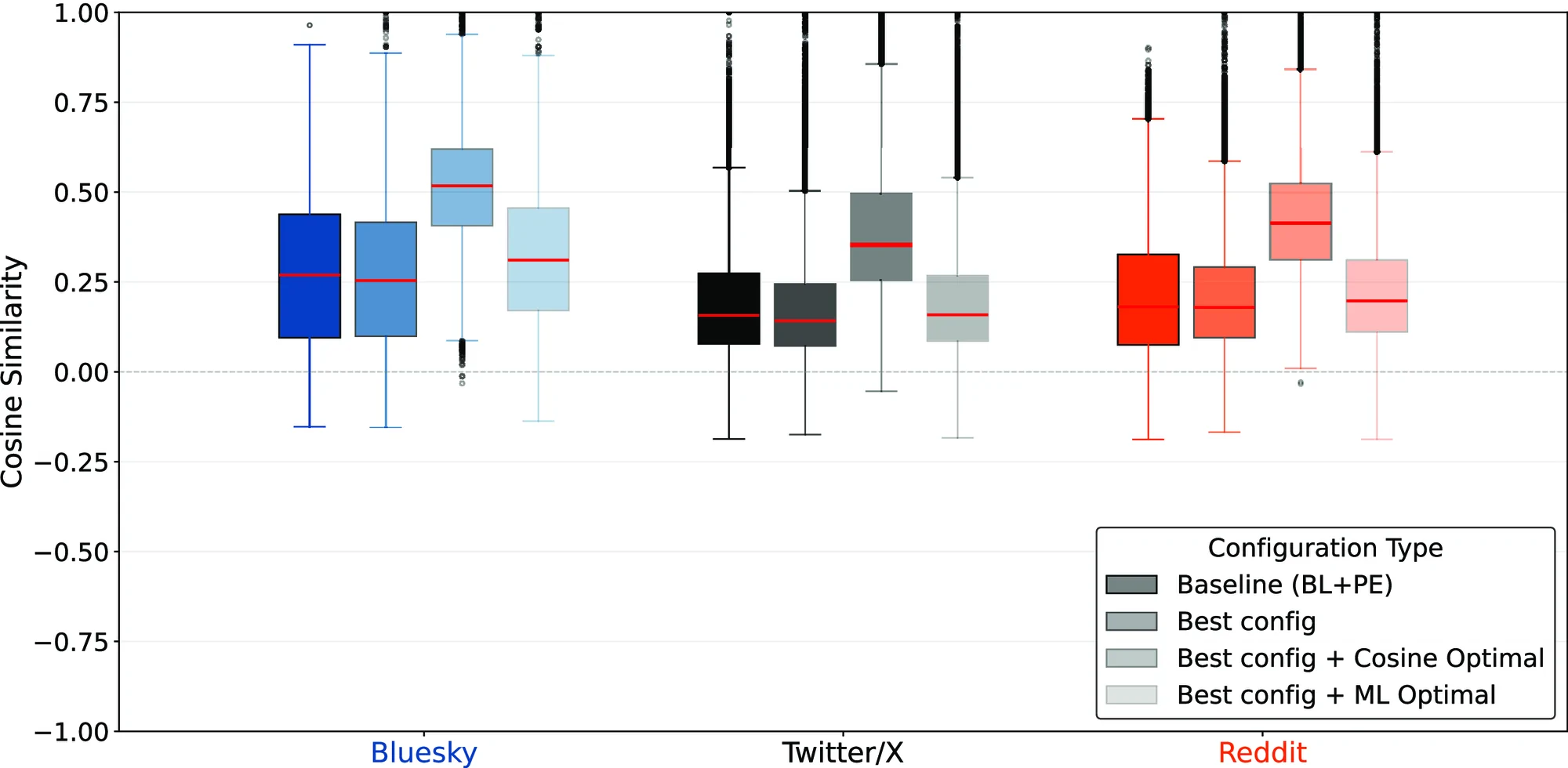
Distribution of cosine similarity scores between AI-generated and ground-truth human replies across all models, comparing four conditions per dataset: reference (BL+PE, darkest), best configuration without selection, best configuration with cosine-optimal selection, and best configuration with ML-optimal selection. A full model/platform-disaggregated version is in *SI Appendix*, Fig. S8.

The relationship between postgeneration selection and semantic similarity reveals unexpected patterns that further illuminate the trade-offs between different optimization objectives (Fig. 11). Most strikingly, cosine-optimal selection substantially increases (roughly doubling) semantic similarity compared to both the baseline and unselected best configurations. For Bluesky, median similarity rises from 0.27 (baseline) to 0.51 (cosine-optimal). Twitter/X shows median similarity increasing from 0.16 (baseline) to 0.35 (cosine-optimal). Reddit exhibits a similar pattern: median similarity climbs from 0.18 (baseline) to 0.41 (cosine-optimal). This demonstrates that when explicitly optimizing for semantic alignment by selecting from 20 candidates, models can produce replies substantially more similar to human ground truth than either reference or best-calibrated configurations.

In contrast, the ML-optimal selection shows rather similar semantic similarity compared to the reference configuration, similar to the results obtained without postgeneration (just using the best model configuration). These values remain substantially below cosine-optimal levels, confirming that optimizing for reduced detectability moves generated content away from the semantic content of human replies.

The stark contrast between cosine-optimal and ML-optimal selection reveals a fundamental tension: the candidate pool of 20 generated replies contains options that are either semantically similar to the reference reply or stylistically optimized to evade detection, but rarely both. When we select for semantic similarity, we can substantially exceed reference configuration performance, achieving median cosine scores of 0.32 to 0.51 across platforms. However, this semantic fidelity comes at the cost of increased detectability, as evidenced by the earlier finding that cosine-optimal selection does not consistently reduce BERT classification accuracy. Conversely, selecting for reduced detectability (ML-optimal) sacrifices semantic alignment, producing replies that sound human-like but say different things than the actual human responses.

This dissociation suggests that current optimal selection strategies, while increasing the diversity of generated candidates, do not enable simultaneous improvement of semantic fidelity and stylistic human-likeness. The candidate space explored by optimized configurations appears to trade off these dimensions: generating more human-like style (which ML-optimal selection exploits) or more semantically aligned content (which cosine-optimal selection exploits), but not both within the same candidate. The ability of cosine-optimal selection to substantially exceed baseline semantic similarity demonstrates that models possess the capacity for greater semantic alignment, but this capacity remains orthogonal to the stylistic characteristics that reduce detectability.

## Discussion

This study provides systematic evidence in the social simulation literature of how far LLMs have come in reproducing human communication, and what specific barriers remain to be addressed. Using social media as a real-world benchmark, we combined a scalable validation framework (which we named CHAT) with a comprehensive calibration benchmark to assess both the realism and the tunability of LLM-generated text. Across three platforms, nine open-weight models, and multiple calibration and postgeneration selection strategies, we found that even the most advanced systems remain distinguishable from human users. Our findings contribute to growing evidence that current LLMs face persistent limitations when used to simulate human behavior across diverse social science applications, from survey research (49) to well-being prediction (50) to political persuasion (11, 51).

### Methodological Contribution and Scope.

Our work builds on the substantial ML/NLP literature on machine-generated text detection, adapting these methods for a fundamentally different purpose: validating whether LLMs can serve as faithful proxies for human participants in social simulations. While detection methods focus on identifying synthetic content, our validation framework emphasizes interpretability, systematic calibration comparison, and platform-specific diagnostics that social scientists need to assess simulation validity. The interpretable feature analysis (Random Forest on stylistic markers, Empath on affective-topical patterns) reveals not just whether LLM text differs from human text, but how—identifying specific dimensions (affective tone, lexical sophistication, platform conventions) where synthetic content systematically diverges. These diagnostics enable researchers to make informed decisions about when and how to use LLMs for behavioral modeling, and highlight the risks of distortion when treating LLM outputs as equivalent to human responses.

### Main Findings.

Calibration and postgeneration selection produce meaningful improvements in human-likeness, but systematic divergences persist and vary predictably across platforms and model types. Classifiers detect AI replies with 70 to 80% accuracy and non-instruction-tuned models such as Llama-3.1-8B and Apertus-8B generally outperform instruction-tuned ones, suggesting that alignment training introduces stylistic regularities that make text more, rather than less, machine-like. Scaling does not help either: the much larger Llama-3.1-70B performs on par with its smaller counterparts. Platform-specific variation further underscores that LLMs reproduce the linguistic “surface” of human communication more easily than its contextual richness: imitation is strongest on Twitter/X, weaker on Bluesky, and weakest on Reddit, where conversational norms are more diverse.

Affective language remains the clearest marker of artificiality. While calibration reduces structural divergences such as sentence length or word count, differences in emotional tone persist. Both toxicity and sentiment consistently emerge as strong discriminators, and topical analyses confirm that emotional expression remains the most resistant dimension of mimicry. LLMs can reproduce the *form* of online dialog but struggle to capture its *feeling*, highlighting a key challenge for future development: calibrating models not only in content but also in emotion, which may require new objectives that explicitly model affective expression rather than relying on stylistic imitation alone. At the same time, our framework offers practical insights for detection and regulation: while synthetic content remains detectable, the gap is narrowing, underscoring the need for continuous monitoring of online discourse as LLM quality improves. Whether this gap eventually closes with future model generations remains an open empirical question that our framework is designed to keep testing; even if it does, however, statistical indistinguishability would be necessary but not sufficient for LLMs to serve as valid behavioral proxies, since matching the surface statistics of human text does not guarantee that a model reproduces the underlying attitudes or reasoning that make human responses meaningful for social-science inference.

Our calibration tests challenge the assumption that more sophisticated prompting strategies necessarily yield more human-like output. Persona descriptions and fine-tuning—strategies often thought to personalize or contextualize LLMs—produce negligible or even adverse effects on realism, whereas simple techniques such as providing stylistic examples or retrieving relevant context consistently reduce detectability. This nonmonotonic pattern shows that greater complexity does not automatically translate to greater realism, and that calibration can introduce new linguistic artifacts even as it helps models adhere to platform norms: after calibration, average word length becomes the dominant feature distinguishing AI from human text, replacing earlier platform-specific cues like hashtags or emoji usage. Rather than eliminating the “signature” of AI authorship, calibration redistributes it across new dimensions of language.

Perhaps our most important finding is the trade-off between semantic fidelity and stylistic human-likeness. Configurations that make LLM text harder to detect simultaneously reduce its semantic similarity to human reference replies, while those that preserve meaning become easier to classify as artificial. This tension holds across all platforms and calibration and selection levels—ML-optimal selection minimizes detectability but sacrifices content accuracy, whereas cosine-optimal selection maximizes semantic alignment but not realism—and extends prior work on competing objectives in LLM optimization (48) by showing that these trade-offs persist even under postgeneration selection. The features that make text *sound* human—stylistic variability, emotional nuance, contextual looseness—are apparently not the same features that make it *mean* what humans mean, suggesting a structural dilemma in which human-likeness and semantic fidelity are competing rather than aligned objectives in current model architectures. This trade-off extends to decoding as well: a targeted ablation (*SI Appendix*, section 12) shows that more permissive decoding lowers detectability, most consistently for ML-optimal selection, but only by monotonically reducing semantic similarity to the human reference reply.

### Implications.

These findings offer concrete guidance for both scientific and applied uses of LLMs. Our benchmark identifies which combinations of model type, calibration strategy, and selection procedure minimize detectability on each platform, giving practitioners a principled basis for design choices. For tasks requiring accurate reproduction of meaning—such as personalized communication or policy simulations—the observed semantic gaps caution against treating LLMs as faithful proxies for human respondents. For applications prioritizing realism—such as synthetic data generation or behavioral modeling—our results identify effective but limited strategies: combining non-instruction-tuned models with stylistic examples, context retrieval, and classifier-based postselection can reduce detectability, but none achieve genuine indistinguishability.

More broadly, this work demonstrates the value of combining scalable quantitative metrics with interpretable linguistic analysis. Our validation framework offers a scalable, reproducible benchmark, while feature-level interpretation reveals where and why models diverge from human language, moving the evaluation of human-like AI from anecdotal judgments toward systematic measurement. By identifying which dimensions of human communication LLMs already approximate well, which respond to targeted calibration, and which remain resistant, our framework gives researchers the tools to make informed, principled decisions about where and how to deploy LLMs in social simulation.

## Materials and Methods

### Data.

For Twitter/X, we build on the dataset by Cerina (42), which contains tweet–reply pairs, tweet metadata, and user-level information; each data point includes a tweet, its parent tweet, and the identity of the replying user. We split the dataset into training and test sets and focus on 275 users with at least 20 replies in the test set, randomly sampling 20 reply tweets per user. Reddit data come from the r/politics subreddit via the Pushshift dataset (44); as with the Twitter/X sample, we selected 492 users and randomly drew 20 reply messages per user following a train–test split. Bluesky data are drawn from the BluePrint dataset (43), a resource for persona-based language modeling curated from the Bluesky platform; we selected 59 user instances, each represented by 20 reply messages after a train–test split—these instances correspond to clusters of similar accounts constructed by the dataset creators rather than to individual users.

### Models.

We evaluated nine open-weight LLMs: apertus-8B-2509 (52), DeepSeek-R1-Distill-Llama-8B (53), Gemma 3 4B Instruct (54), Llama 3.1 8B (55), Llama 3.1 8B Instruct (56), Llama 3.1 70B (57), Mistral 7B v0.1 (58), Mistral 7B Instruct v0.2 (58), and Qwen 2.5 7B Instruct (59). Each model was run with temperature T=0.8, nucleus sampling (P=0.9), and top-k sampling (k=50); these are, individually, widely used defaults for open-ended sampling—top-P=0.9 is the representative setting from work that introduced nucleus sampling (60), and top-k=50 is the default value in the transformers generation configuration used for all our generation—and combining all three constrains the sampling distribution on all three axes simultaneously, compounding their entropy-reducing effect beyond what any single one would produce alone; we characterize the consequences of this choice directly via a decoding hyperparameter ablation (*SI Appendix*, section 12). Prompts included a persona description generated by Llama-3.1-8B-Instruct based on up to 30 user messages randomly sampled from each user’s training set, adapted for each platform; full prompt specifications, including persona generation, are provided in *SI Appendix*, sections 1 and 2).

### Detectability.

We pair each AI-generated reply with the corresponding human ground-truth reply to the same parent message, creating a balanced dataset where AI and human replies address identical conversational contexts. The classifier receives only the reply text as input, not the parent message being replied to, ensuring that classification is based solely on intrinsic linguistic characteristics (style, tone, word choice, structure) rather than on topical or contextual differences. We train the BERT-based sequence classification model to discriminate between AI-generated and human-authored text, evaluating its performance on a held-out validation set (80/20 train-validation split); to ensure statistical robustness and minimize overfitting risks, we repeat this procedure three times using different random seeds and report the average accuracy (with SD) across runs. Because all configurations are evaluated on the same pool of reply pairs, these comparisons are best interpreted as descriptive benchmarking of relative detectability within our experimental setup rather than as a strict out-of-sample model selection procedure.

### Semantic Similarity.

We embed each reply into a dense vector space using the all-MiniLM-L6-v2 model from the SentenceTransformers library (61). This sentence embedding model (22.7M parameters, 384-dimensional embeddings) was trained using contrastive learning on over 1 billion sentence pairs from diverse sources, including Reddit comments, making it well-suited for measuring semantic similarity in social media contexts independent of surface-level lexical overlap. For each human-authored/AI-generated reply pair, we compute the cosine similarity between their embeddings and analyze the distribution of these scores across all reply pairs.

### Interpretable Features.

We train a Random Forest classifier on interpretable textual indicators capturing lexical, stylistic, affective, and structural dimensions of online conversation: lexical/structural features—word count, character count, average word length, sentence length variance, punctuation count, uppercase count, mention count, hashtag count, link count, emoji count, transition word count, superlative count, and hedge word count (based on a predefined lexicon); *diversity and repetitiveness*—type–token ratio (lexical diversity), 3-g repetition count, and a proxy for perplexity (self-entropy of the word distribution); *stylistic/affective features*—sentiment polarity scores (via VADER) (62) and toxicity score (using the unitary/toxic-bert model) (63); syntactic complexity—sentence count and average clauses per sentence, approximated by the number of commas plus one. Separately, we employ the Empath library (45), which identifies and quantifies the presence of 194 built-in topical categories (e.g., emotions, social relationships, cognitive processes) by analyzing lexical patterns within the text; for each reply we extract normalized topic signal scores across all categories, compare AI-generated and human-authored distributions per category using the Wilcoxon rank-sum test, apply Benjamini–Hochberg false discovery rate (FDR) correction across the 194 categories, and identify features as significantly different when the adjusted P-value is below 0.05.

### Calibration and Postgeneration Selection.

Five calibration configurations of increasing complexity (baseline, persona, +stylistic examples, +context retrieval, +fine-tuning) are applied across all nine models and three datasets; fine-tuning uses the Parameter-Efficient Fine-Tuning (PEFT) library (47) on batches of up to 60 users containing between 80 and 200 training messages per user (*SI Appendix*, section 1). For postgeneration selection, each model produces 20 replies per test message: the Cosine Similarity Selection procedure selects the candidate with the highest cosine similarity to the ground-truth reply, while the ML-Optimal Selection procedure trains a fold-wise Random Forest classifier (100 estimators, fixed random seed) on the same interpretable feature set to estimate each candidate’s likelihood of being misclassified as human, selecting the reply with the highest misclassification probability.

## Supplementary Material

Appendix 01 (PDF)

## Data Availability

Anonymized social media data and processing code have been deposited in Zenodo (https://zenodo.org/records/21891179) (64). Previously published data were used for this work. Twitter/X data were collected using the official Twitter API, starting from the user list originally compiled by Cerina (42). Reddit data were obtained from the r/politics subreddit via the Pushshift dataset (44). Bluesky data were drawn from the BluePrint dataset (43). This study was approved as exempt by the Human Subjects Committee of the Faculty of Business, Economics and Informatics at the University of Zurich (OEC IRB # 2026-012). To protect user privacy, all data were anonymized by replacing usernames with hashed identifiers and anonymizing in-post mentions, except for 15 accounts identified as public figures (defined as having at least 1 million followers on Twitter/X). All computational analyses were performed using locally deployed models on the University of Zurich Science Cluster, so no user data were transmitted to external services or third-party APIs at any time.
