# Supplementary material for: AI-generated language remains systematically distinguishable from human communication
Source: Proc Natl Acad Sci U S A. 2026 Oct 12;123(42):e2531748123. doi: 10.1073/pnas.2531748123 (PMC13684137; doi:10.1073/pnas.2531748123)
Supplement: Supplementary file 1 [file pnas.2531748123.sapp.pdf]

Supporting Information for

AI-Generated Language Remains Systematically Distinguishable from Human Communication

Nicolò Pagan, Petter Törnberg, Christopher A. Bail, and Christopher Barrie

Nicolò Pagan.
E-mail: nicolo.pagan@uzh.ch

This PDF file includes:

Supporting text
Figs. S1 to S12
Tables S1 to S3
SI References

Supporting Information Text

1. Complete Prompt Specifications

This section provides the complete prompt templates and generation parameters used in our experiments, enabling exact reproduction of our results.

A. Persona Generation. All personas were generated using Llama-3.1-8B-Instruct with the following configuration:

- Temperature: $T = 0.4$
- Top-p (nucleus sampling): $p = 0.9$
- Maximum new tokens: 300
- Input: Up to 30 training messages per user (sampled to ensure diversity)

A.1. Persona Generation System Prompt.

```
You are a social media researcher who writes concise persona descriptions.

CRITICAL RULES:
1. Write in THIRD PERSON only (e.g., "They are...", "This user tends to...")
2. DO NOT copy, quote, or paraphrase any posts
3. DO NOT use bullet points or lists
4. DO NOT ask questions
5. Write 2-3 flowing paragraphs describing personality, interests, and
   communication style
6. Focus on patterns and traits, not specific post content
```

A.2. Persona Generation User Prompt. For each platform, we used the following template, adapting the user prefix to the platform, using @ for Twitter/X and Blueksy, and u/ for Reddit.

```
Example of a GOOD persona description:
@ExampleUser is a politically engaged individual with progressive views
and a strong interest in social justice issues. They communicate in a
direct, sometimes passionate tone, often sharing news articles and
commentary on current events.

Their writing style is informal but articulate, frequently using humor
and sarcasm to make points. They appear well-informed about policy
matters and enjoy engaging in substantive debates. They show particular
interest in environmental issues, workers' rights, and democratic processes.

Overall, @ExampleUser comes across as an active community member who
values informed discourse and isn't afraid to voice strong opinions
while remaining open to discussion.

---

Now analyze this {platform} user. Based on their posts below, write a
similar persona description starting with "{user_prefix}{username} is".

Remember: NO bullet points, NO quotes from posts, NO first person,
just flowing paragraphs describing who this person is.

Posts from {user_prefix}{username}:
1. {post_1}
2. {post_2}
...
{up to 30 posts}

Persona description for {user_prefix}{username}:
```

A.3. Persona Format Transformation. Generated personas were in third person (e.g., "@username is a politically engaged individual..."). For instruction-tuned models, we programmatically transformed these to second person using Llama-3.1-8B-Instruct with temperature $T = 0.1$ and nucleus sampling ($p = 0.95$).

Transformation System Prompt:

```
You are a text transformation assistant. Your task is to rewrite
persona descriptions from third person to second person.

Rules:
1. Change "@username is" or "u/username is" to "You are @username,"
   or "You are u/username,"
2. Change "They" to "You"
3. Change "Their" to "Your"
4. Change "their" to "your"
5. Change "they" to "you"
6. Change "them" to "you"
7. Change "themselves" to "yourself"
8. Keep the content exactly the same, only change the perspective
9. The result should address the user directly in second person
10. Output ONLY the transformed text, no explanations or additional text
```

Transformation User Prompt:

```
Transform this persona description from third person to second person:

Original (third person):
{persona_third_person}

Transformed (second person, addressing the user directly as "You"):
```

The final dataset structure contained two persona columns:

- `persona`: Second person format (for instruction-tuned models)
- `persona_third_person`: Third person format (for non-instruction-tuned models)

B. Response Generation. All response generation used the following parameters:

- Temperature: $T = 0.8$
- Top-p (nucleus sampling): $p = 0.9$
- Top-k sampling: $k = 50$
- Maximum new tokens: 300
- Do sample: True
- Repetition penalty: 1.0

B.1. Instruction-Tuned Model Prompts. For instruction-tuned models (Llama-3.1-8B-Instruct, Mistral-7B-Instruct-v0.2, etc.), we used the following template structure:

```
[Instruction]
{persona_second_person} # Only for PE configuration, else "You are {user_prefix}{username}"

[Writing Style] # Only for PE+SE configuration
These are some posts that represent how you, {user_prefix}{username}, write:
- {example_1}
- {example_2}
...
- {example_n}

[User Retrieved Context]  # Only for PE+SE+CR configuration
This is some useful context retrieved from {user_prefix}{username}'s history:
{retrieved_context}

[Conversation]
Here is a conversation in which you, as {user_prefix}{username}, are participating.
{parent_post}
```

```
[Instruction]
Continue the conversation naturally by adding {user_prefix}{username}'s reply of a length which is appropriate
for {platform}.
```

We adapt the user prefix to the platform, using @ for Twitter/X and Blueksy, and u/ for Reddit.

B.2. Non-Instruction-Tuned Model Prompts. For base models (Llama-3.1-8B, Mistral-7B-v0.1, etc.), we used completion-style prompts:

```
{Platform} conversation with {user_prefix}{username}

About {user_prefix}{username}: {persona_third_person} # Only for PE configuration

Example posts by {user_prefix}{username}: # Only for PE+SE configuration
- {example_1}
- {example_2}
...
- {example_n}

Relevant context from {user_prefix}{username}'s history:  # Only for PE+SE+CR configuration
{retrieved_context}

Conversation:
{parent_post}
{user_prefix}{username}:
```

Similarly, we adapt the user prefix to the platform, using @ for Twitter/X and Blueksy, and u/ for Reddit.

C. Fine-Tuning Configuration. For fine-tuned models, we used LoRA (Low-Rank Adaptation) with the following parameters:

- Rank: $r = 16$
- Alpha: $\alpha = 32$
- Dropout: 0.1
- Target modules: q_proj, v_proj, o_proj, gate_proj, up_proj, down_proj
- Training epochs: 1 (with early stopping, patience=3)
- Learning rate: 10^{-4} with cosine scheduler
- Batch size: 2 per device, gradient accumulation steps: 4 (effective batch size: 8)
- Maximum sequence length: 256 tokens
- Maximum training examples per user: 200 (randomly sampled)

Fine-tuning data format:

```
You are {user_prefix}{username}. Respond to the following {tweet / Reddit post/ Bluesky post}:
Tweet / Reddit post / Bluesky post: {parent_post}
Response: {user_reply}<eos>
```

Labels were masked for the prompt portion (up to "Response: "), so loss was only computed on the actual user reply.

D. Fine-Tuning Data. Fine-tuning is performed per batch of up to 60 users jointly: a single model is adapted on the combined training messages of all users in the batch, up to a maximum of 200 messages per user (randomly sampled). Users with fewer than 100 total posts are excluded from fine-tuning, yielding between approximately 80 and 200 training messages per user. The training data is split 90/10 into train and validation sets; the 10% validation split is used for early stopping (patience = 3 evaluations, evaluated every 200 steps).

2. Response cleaning

LLM-generated replies were passed through a deterministic cleaning step before the analysis. The cleaner addresses a systematic class of generation artifact: several models prepend a formatting prefix to their output that does not belong to the reply itself. These prefixes fall into two broad families — *handle prefixes*, where the model echoes the simulated user's own handle at the start of the reply, and *bracket/label prefixes*, where the model outputs an instruction-leaked or template-inherited header such as [Output], [Reply], or Response:. Such artifacts arise because the prompt delivered to each model contains the target user's handle and a structured instruction block; some models reproduce parts of this context verbatim before generating the actual reply.

Cleaning rules. We define eight rules applied sequentially to each response string.

@handle prefix. A leading @User_XXXX: or @anon_user_XXXX: prefix (including bold-wrapped variants such as **@User_XXXX:**) is stripped when the handle matches the simulated author of the reply. References to *other* users' handles (e.g., @ElizabethScudder:) are left intact. If the same handle appears twice consecutively (a rare double-echo artifact), both occurrences are removed.

Reddit u/-handle prefix. A leading u/User_XXXX: or u/anon_user_XXXX: prefix, including bold-wrapped variants, is stripped under the same author-match condition.

Bare user prefix. A bare User_XXXX: prefix without the leading @ is stripped under the same author-match condition.

[Output] prefix. The literal prefix [Output] (case-insensitive) is removed.

Response-label prefix. Bare response-label prefixes of the form Response:, Reply:, **Response:**, or **Reply**: are stripped (case-insensitive, with optional my qualifier).

Generic bracket prefix. Any remaining [tag] or [tag]: prefix is removed when non-whitespace content follows, covering patterns such as [Reply], [Response], or [Instruction].

Bracket-only discard. If the entire response consists of bracket tags ([...]) or bold formatting (**...**) with no substantive content interspersed, the response is discarded. This covers outputs such as [Conversation], [Response], or **Please respond to the user.** that contain nothing beyond leaked instruction tokens. This rule is checked both before and after prefix-stripping, to catch cases where stripping a handle reveals an otherwise empty string.

<|endoftext|> removal. The <|endoftext|> special token, a Qwen artifact, is removed wherever it occurs (leading, trailing, or mid-response). A residual Human: prefix left after token removal is also stripped.

Entry-level invariants. Cleaning is applied to both the selected response and the full list of candidate responses stored for each simulated turn. If the selected response cleans to an empty string but other candidates remain, the first non-empty candidate is promoted as the selected response. If all candidates clean to empty, the entire entry is dropped from the dataset.

3. Feature Set for ML-Based selection

To train the Random Forest classifier used in the ML-based classification procedure, we extract a diverse set of interpretable features from each candidate reply. These features are designed to capture lexical, stylistic, affective, and structural properties of online discourse. Table S1 summarizes the full feature set employed in both the interpretable analysis and the ML-optimal selection procedure.

Table S1. Detailed overview of features used in the ML-based selection procedure.

Dimension	Feature description
Lexical/structural	Word count (number of tokens); Character count (total characters in reply); Average word length (characters per word); Sentence length variance (variance of words per sentence); Sentence count (number of sentences); Punctuation count (number of punctuation marks); Uppercase count (uppercase characters); Mention count (number of @user mentions); Hashtag count (number of #hashtags); Link count (number of URLs); Emoji count (number of emojis); Transition word count (discourse markers such as "however," "therefore"); Superlative count (e.g., "best," "most important"); Hedge word count (predefined hedging terms such as "perhaps," "might," "likely").
Diversity/repetitiveness	Type–token ratio (lexical diversity); Repetitive patterns (count of repeated 3-grams); Perplexity proxy (self-entropy of word distribution).
Stylistic/affective	Sentiment polarity (VADER); Toxicity score (predicted using unitary/toxic-bert); Abstract–concrete ratio (balance between abstract and concrete terms).
Syntactic complexity	Average clauses per sentence (approximated by number of commas + 1).

4. Post-Generation Selection Method Overlap

Figure S1 analyzes the overlap between different response selection methods, averaged across all configurations for each model and dataset. We compare three selection approaches: the first generated response (no selection), ML-optimal selection (highest misclassification probability), and cosine-optimal selection (highest semantic similarity to reference reply). The bars show the percentage of test cases where different selection methods chose the same reply.

Overlap rates between ML-optimal and cosine-optimal selection remain consistently low across models and datasets, typically ranging from 5-10%. This barely exceeds the 5% random chance baseline (1 out of 20 candidates), confirming that these selection methods optimize for fundamentally distinct characteristics and identify different replies from the candidate pool. Similarly, the overlap between either selection method and the first response hovers around 5-10%, indicating that both selection procedures consistently prefer alternatives to the default output.

One notable exception appears with gemma-3-4b-it, which shows elevated overlap rates reaching 20-30% across various comparisons. This suggests reduced diversity in the generated candidate pool for this model, potentially indicating that some of the 20 generated replies are exact duplicates rather than distinct alternatives. For most models, however, the near-baseline overlap rates demonstrate that the 20 generated candidates exhibit substantial diversity, and that ML-optimal and cosine-optimal selection exploit this diversity in orthogonal ways, reflecting their different optimization objectives.

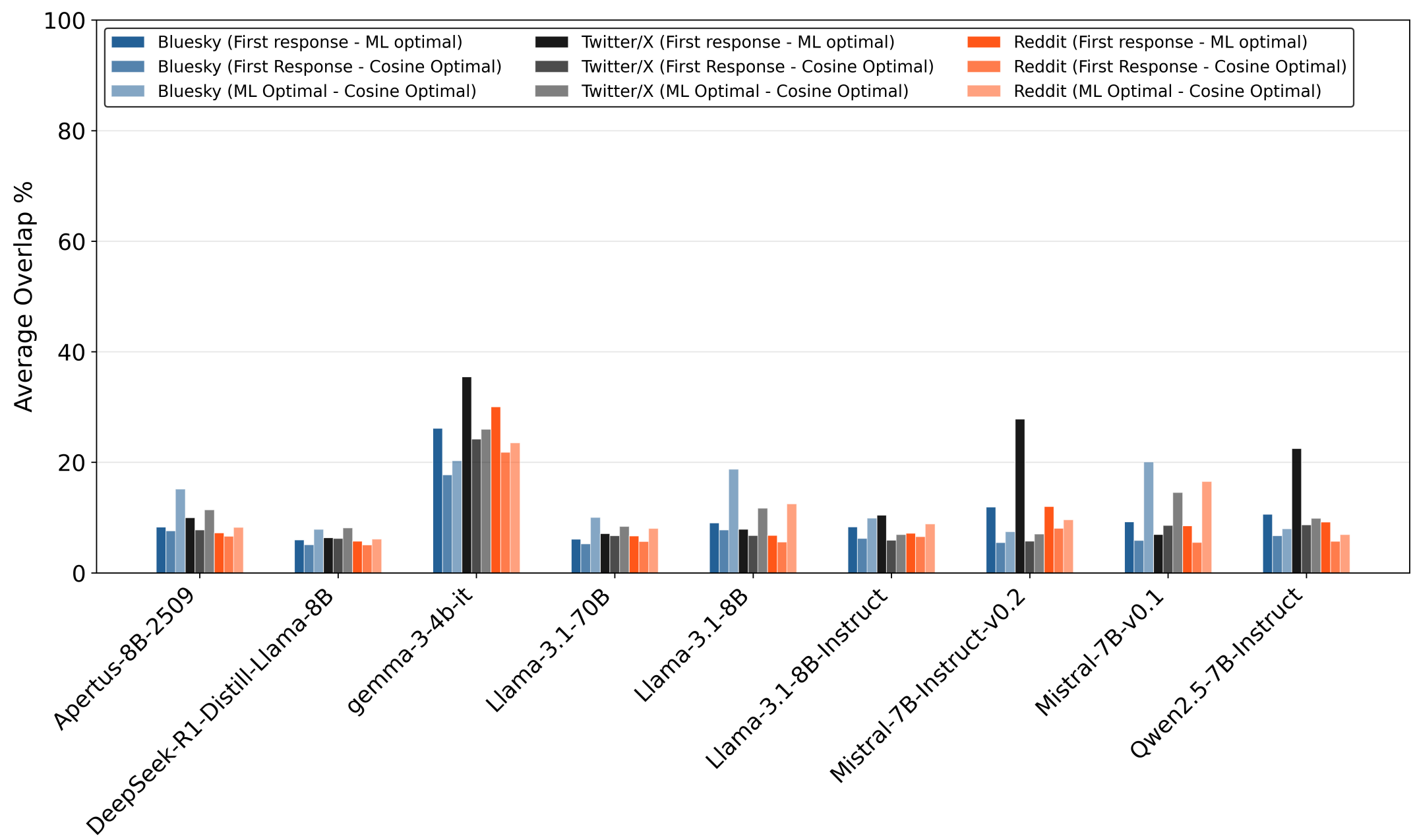

Fig. S1. Overlap between response selection methods averaged across all configurations for each model and dataset. Three comparisons are shown: First response vs. ML-optimal (how often ML-optimal selects the first generated reply), First response vs. Cosine-optimal (how often cosine-optimal selects the first generated reply), and ML-optimal vs. Cosine-optimal (how often both methods select the same reply). Overlap rates consistently hover around 5-10%, barely exceeding the 5% random chance baseline, confirming that the two selection methods target distinct reply characteristics. Elevated overlap for gemma-3-4b-it suggests reduced candidate diversity for this model.

5. Per-platform and per-model Cosine Similarity Analysis (reference configuration results)

Figure S2 reports the distribution of cosine similarity between each AI-generated response and the corresponding ground-truth human response, broken down by model and platform. All results use the reference configuration, namely baseline with persona description only, with no fine-tuning, style examples, or context retrieval. Each boxplot aggregates similarity scores over all users in the dataset for the given model–platform pair. A higher cosine similarity indicates that the generated response is semantically closer to what the human actually wrote in reply to the same prompt. Results demonstrate that models perform somewhat consistently, with the notable exceptions of Mistral-7B-v0.1 and Llama-3.1-8B on Bluesky and Reddit, performing particularly poorly (barely positive median cosine similarity score).

6. Per-model feature importance (reference configuration results)

Figure S3 disaggregates the platform-averaged feature importance heatmap by model. Two broad clusters emerge, broadly aligned with the base/instruction-tuned divide.

Base models: length and format as primary discriminators. For Mistral-7B-v0.1 and Llama-3.1-8B, the dominant signal is *uppercase ratio* (MDI = 0.196 and 0.134, respectively). Both models also show high importance for *perplexity proxy* and *word count*, confirming that length and vocabulary diversity are sufficient to distinguish their outputs from human replies.

 Nicolò Pagan, Petter Törnberg, Christopher A. Bail, and Christopher Barrie

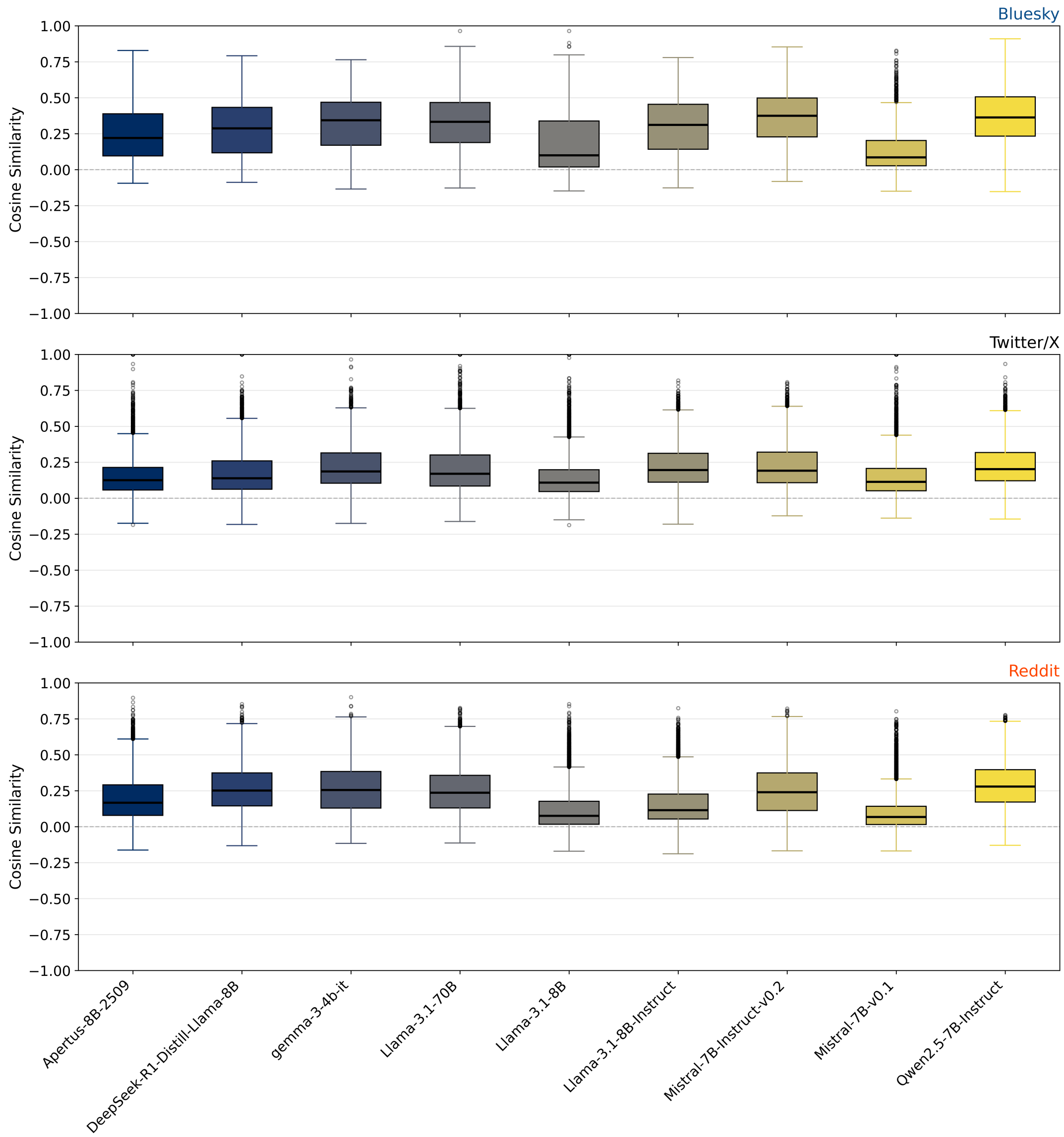

Fig. S2. Distribution of content similarity between the AI-generated response and the ground-truth response, measured via the cosine similarity score using the `all-MiniLM-L6-v2` model from the `SentenceTransformers` library, and disaggregated by platform and model. The plot refers to the reference configuration, with no post-generation selection optimization.

LLAMA-3.1-70B exhibits a more balanced profile across these same features with no single outlier, consistent with its stronger baseline performance.

Instruction-tuned models: social signals dominate. The instruction-tuned models tell a markedly different story. For MISTRAL-7B-INSTRUCT-V0.2, *mentions count* (MDI $= 0.199$) and *hashtags count* (0.131) are by far the most discriminative features. QWEN2.5-7B-INSTRUCT similarly places heavy weight on *hashtags count* (0.122) and *perplexity proxy* (0.126). These results suggest that instruction tuning causes models to reproduce conversational writing conventions (tagged topics) that are largely different from those in the target social media reply register.

Specialised models. GEMMA-3-4B stands out for the high importance of *sentence count* (0.118) and *punctuation count* (0.113), reflecting its tendency to produce longer, structurally more complex replies with multiple punctuated clauses (see next section). DEEPSEEK-R1-DISTILL-LLAMA-8B shows elevated importance for *quotes* (0.103). APERTUS-8B presents a relatively flat profile, with no single feature far above the rest, which aligns with its relatively strong aggregate performance.

Shared discriminators. Across all models, *perplexity proxy* and *word count* appear with non-negligible importance (MDI ≥ 0.09 for most models), confirming their status as platform-agnostic discriminators between human and AI text. Features in the right-hand tail of the heatmap—*superlatives*, *abstract–concrete ratio*, and *transition words*—contribute consistently near zero for all models, indicating that these higher-level semantic dimensions are not exploited by the classifier under this configuration.

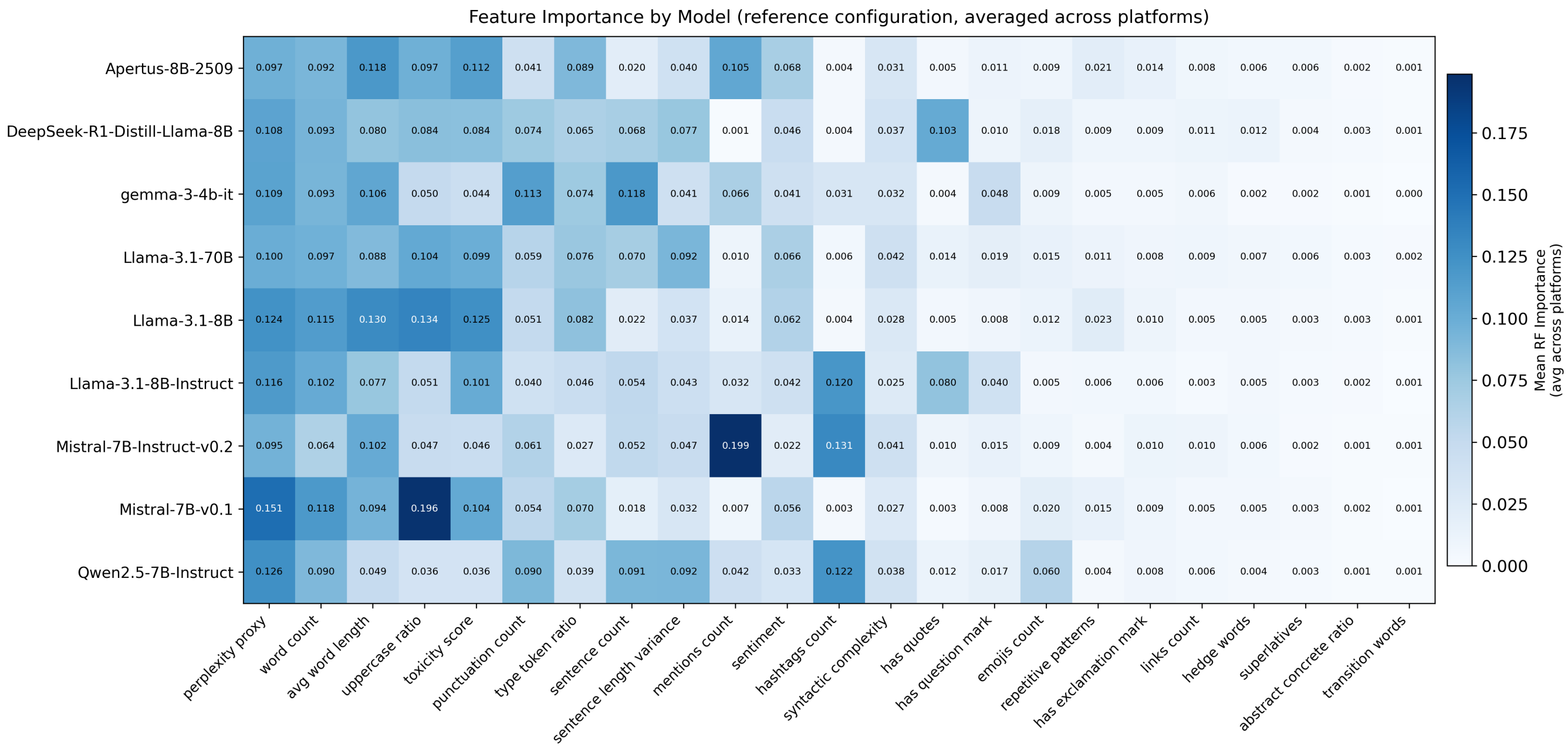

Fig. S3. Mean Decrease in Impurity (MDI) feature importance for each of the nine models under the reference configuration (no fine-tuning, no persona, no context), averaged across all three platforms. Each cell reports the MDI weight of a given feature (columns) for a given model (rows); darker colours indicate higher discriminative contribution. The rightmost colour-bar axis reports the scale in average MDI units.

A. Feature importance: MDI vs. permutation. The feature importance rankings used throughout the previous analysis are based on Mean Decrease in Impurity (MDI), a standard byproduct of Random Forest training. MDI measures how much each feature reduces Gini impurity across all splits and trees, and is computed on the training data. It is known to be biased toward high-cardinality continuous features, such as word count, which offer many possible split thresholds and are therefore selected more often during tree construction, independently of their true predictive value.

As a robustness check, Figure S4 compares MDI against Permutation Feature Importance (PFI) for each platform. PFI is computed on a held-out test set: each feature is shuffled in turn, and the resulting drop in AUC is recorded, giving an out-of-sample estimate of predictive contribution that is not inflated by training-set overfitting.

The comparison shows that the two methods yield broadly consistent rankings. The most discriminative features, namely mentions count, hashtags count, toxicity score, uppercase ratio, and average word length, appear prominently under both criteria across all three platforms. The main discrepancy is that word count and perplexity proxy rank somewhat higher under MDI than under PFI, consistent with the expected high-cardinality inflation. Conversely, uppercase ratio and toxicity score rank higher under PFI, suggesting their predictive contribution is slightly underestimated by MDI. These shifts are incremental rather than reversals: no feature that ranks in the top 10 under MDI is absent from the top 10 under PFI. We therefore retain MDI as the primary importance measure throughout the paper, noting that the qualitative conclusions are robust to this methodological choice.

7. Per-platform and per-model feature distribution differences (reference configuration results)

Figure S5 examines how AI-generated content differs from human-authored content along interpretable linguistic dimensions. For each platform, we identify the five features with the highest mean importance in the Random Forest classifier (averaged across models), which vary across platforms as different stylistic cues are most discriminative in each social media context. For each model, we display the distribution of raw feature values for AI-generated responses as a boxplot, alongside the distribution for human-authored content (gray). The dashed horizontal line marks the human median for that feature, serving as a reference for what a typical human response looks like. Models whose distributions are consistently above or below this line systematically over- or under-produce that feature relative to human writing. Results are shown under the reference configuration, split by platforms and models. We discuss each feature group in turn.

Word count. Human replies are moderately long on Bluesky (33 words), short on Twitter (median 9 words), and intermediate on Reddit (16 words), Most models overshoot human verbosity. The most verbose models are Gemma-3-4b (median 65 words on Bluesky, 39 on Twitter) and Llama-3.1-8B-Instruct (53 on Bluesky, 40 on Twitter), which exceed human length by roughly a factor of two. Two models stand as exceptions in the opposite direction: Llama-3.1-8B produces very short outputs (median 3–6 words across platforms) and Mistral-7B-v0.1 nearly always outputs a single token or very few words (median 1–3 words), collapsing to near-empty replies. This verbosity mismatch suggests that without explicit length guidance, instruction-tuned models default to a more formal, elaborate register than actual social media users, while pure base models

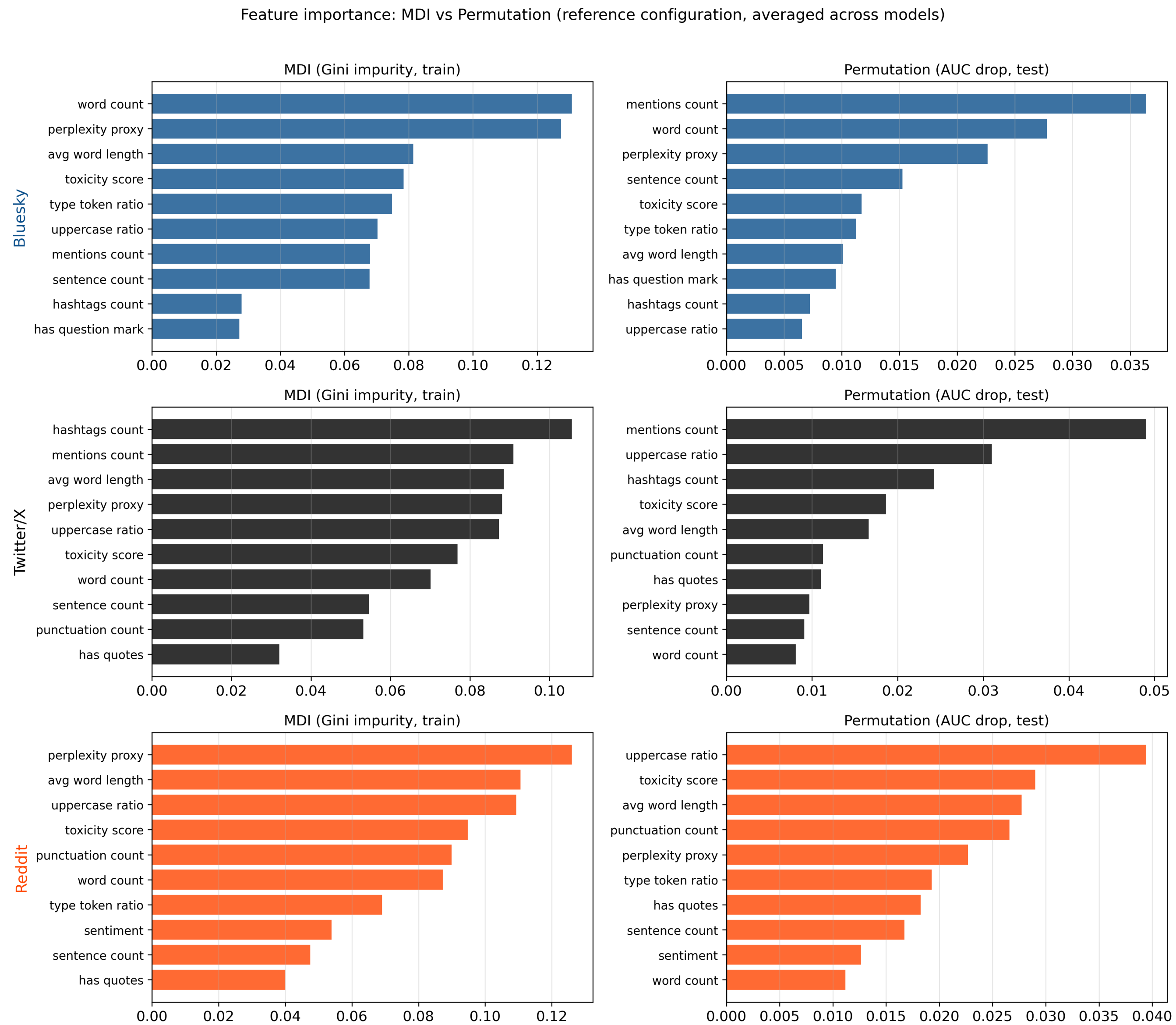

Fig. S4. Comparison of Mean Decrease in Impurity (MDI, left column) and Permutation Feature Importance (PFI, right column) for each platform (rows), averaged across all nine reference-configuration models. MDI is a byproduct of Random Forest training and is known to inflate high-cardinality continuous features; PFI measures the drop in AUC when each feature is shuffled on a held-out test set, providing an unbiased out-of-sample estimate. The rankings are broadly consistent across methods; the main differences are that features like word count and perplexity proxy are somewhat inflated by MDI, while uppercase ratio and toxicity score rank higher under PFI.

generate degenerate short continuations unsuited to the reply task.

Average word length. This feature is largely consistent across models and platforms, clustering around the human reference value of 4.6–4.8 characters per word. LLAMA-3.1-8B shows a systematic downward deviation (3.7–4.0), reflecting its simpler vocabulary, consistent with its tendency toward very short outputs. At the other end, MISTRAL-7B-INSTRUCT-V0.2, QWEN2.5-7B-INSTRUCT, and GEMMA-3-4B slightly exceed human average word length on all three platforms, suggesting a marginally more formal or technical lexical register.

Perplexity proxy (vocabulary entropy). This feature measures Shannon entropy over word frequencies within a text: $H = -\sum_w p(w)\log_2 p(w)$, where $p(w)$ is the relative frequency of word w. It captures within-text vocabulary diversity independently of length—a short text where every word is distinct yields entropy equal to $\log_2 n$ (where n is word count), while repetitive or degenerate outputs collapse toward zero. MISTRAL-7B-V0.1 produces values near 1.0 across all platforms, indicating near-degenerate outputs with almost no vocabulary variation. LLAMA-3.1-8B similarly scores 1.6–2.8, well below the human range of 3.3–5.0. Most other models roughly match or slightly exceed human entropy, suggesting adequate within-text lexical diversity once replies are long enough; the remaining gap is largely attributable to the length differences discussed above.

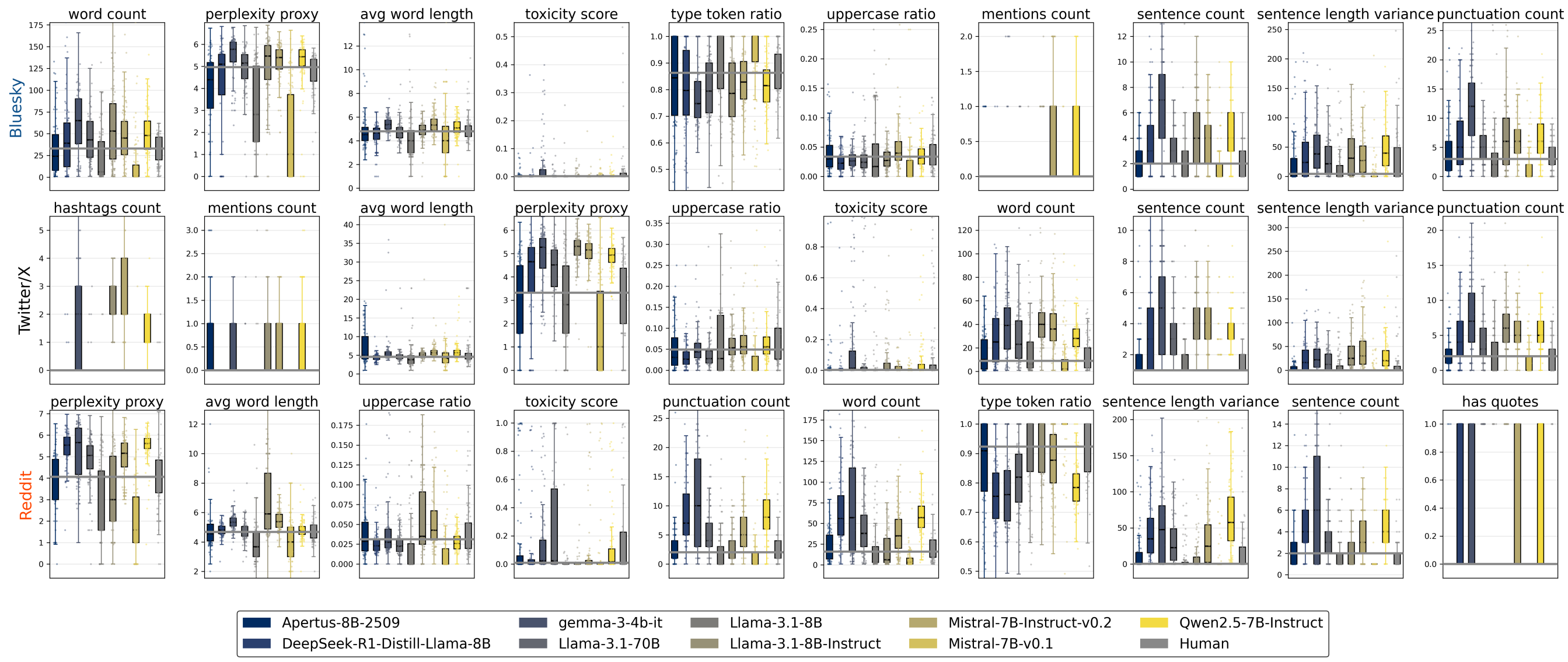

Fig. S5. Raw feature distributions of AI-generated content and human-authored content, split by model and platform. For each platform, the top 5 most important features (averaged across models) are shown. The gray boxplot represents human-authored content; the dashed line marks the human median.

Toxicity. Toxicity scores are highly right-skewed: most replies score near zero, with a long tail of flagged content. The feature is therefore better compared via means than medians. Human mean toxicity is highest on Reddit (0.20), reflecting the platform's more confrontational discourse norms, and lower on Twitter (0.11) and Bluesky (0.06). *The majority of models underestimate toxicity on all platforms*, particularly safety-aligned instruction-tuned models: LLAMA-3.1-8B-INSTRUCT scores 0.021 on Reddit (ten times below the human mean), and MISTRAL-7B-INSTRUCT-V0.2 scores 0.070 (three times below). MISTRAL-7B-V0.1 and LLAMA-3.1-8B likewise generate almost no toxic content. Two models partially reverse this trend: on Reddit, LLAMA-3.1-70B slightly *overestimates* toxicity (0.268 vs. human 0.203), as does GEMMA-3-4B on Twitter/X (0.134 vs. human 0.110). On Bluesky, however, even these models fall below the human reference. The overall pattern suggests that safety training suppresses toxic content to a degree that does not match the residual toxicity naturally present in social media discourse.

Hashtag usage. Human hashtag usage is sparse: the median is zero on all platforms, and the proportion of messages containing at least one hashtag is only 3% on Twitter, 9% on Bluesky, and 0.6% on Reddit. Most base models respect this sparsity (APERTUS-8B, DEEPSEEK-R1-DISTILL-LLAMA-8B, LLAMA-3.1-8B, MISTRAL-7B-V0.1: ≤4% on Twitter). Instruction-tuned models, by contrast, dramatically overuse hashtags. On Twitter, LLAMA-3.1-8B-INSTRUCT includes at least one hashtag in 91.5% of its replies, MISTRAL-7B-INSTRUCT-V0.2 in 85.7%, and QWEN2.5-7B-INSTRUCT in 83.4%, inflating a near-zero feature by two orders of magnitude. The same pattern holds on Bluesky (QWEN2.5-7B-INSTRUCT: 53%, MISTRAL-7B-INSTRUCT-V0.2: 41%) and, to a lesser extent, on Reddit (MISTRAL-7B-INSTRUCT-V0.2: 25%, QWEN2.5-7B-INSTRUCT: 27%), where hashtags are almost entirely absent from human text. LLAMA-3.1-70B is the only large instruction-tuned model that behaves more conservatively, staying below 10% on all platforms. This systematic overuse likely reflects training on instructional or web-scraped text where hashtags signal topic emphasis, a convention absent in human reply behavior.

Mention usage. Human replies contain virtually no @mentions on Twitter (0.04% of texts, as Twitter stores the reply-target as metadata rather than embedding it in the message body), and fewer than 0.3% on Bluesky and Reddit. Base models broadly replicate this near-zero rate. Instruction-tuned and larger models, however, frequently hallucinate @mentions in contexts where none are warranted. The most extreme cases on Twitter are MISTRAL-7B-INSTRUCT-V0.2 (62.6% of replies contain at least one mention), APERTUS-8B (50.6%), and QWEN2.5-7B-INSTRUCT (45.0%). On Bluesky, MISTRAL-7B-INSTRUCT-V0.2 is a stark outlier at 67.5%, compared to a human rate of 0.3%. On Reddit, mention usage remains near zero for all models, consistent with the human reference. These hallucinated mentions likely stem from training exposure to conversational formats (e.g. threaded discussions or instruction-tuning corpora) where addressing interlocutors by handle is common, a pattern models incorrectly generalize to the reply-generation task.

Summary. Taken together, the feature-level analysis reveals two broad failure modes. First, a *systematic safety bias*: instruction-tuned models suppress toxicity well below human levels, creating a consistent gap. Second, a *format hallucination bias*: the same models overuse platform-specific social signals (particularly hashtags and mentions) that are largely absent in human replies, suggesting that model behavior encodes writing conventions from training data genres that do not match the reply register. Base models avoid both of these biases but introduce their own failure mode: degenerate or near-empty outputs with near-zero vocabulary diversity.

8. Cosine Similarity Baselines

To contextualise the cosine similarity between AI-generated responses and human ground-truth messages (denoted *human-vs-AI*), we compute a set of reference distributions that span the range from semantically unrelated random text pairs to near-identical responses generated from the same prompt, including a same-context human baseline (different users replying to the same source post) that calibrates how much of the AI–AI and human-vs-AI similarity reflects shared context rather than shared authorship. All similarities are computed with the `all-MiniLM-L6-v2` sentence-transformer model, which maps texts to ℓ_2-normalised 384-dimensional embeddings; cosine similarity therefore reduces to the inner product of two embedding vectors.

Data scope. The main distributional analysis (Figure S6) uses the reference configuration: baseline (no fine-tuning), no additional context (`ctx0`), no style conditioning (`style0`), with persona. This yields 9 AI models per platform (one reference configuration file each), producing 9,783 test entries for Bluesky, 87,226 for Reddit, and 47,701 for Twitter. Each entry contains (i) the human ground-truth message (`original_message`), (ii) a single selected AI response (`response`), and (iii) up to 20 candidate responses from the same model and prompt (`all_valid_responses`). The human training corpus used for the intra-human and random-human baselines comprises all training-split messages from `posts.pkl`: 35,914 messages from 60 users (Bluesky), 159,273 messages from 492 users (Reddit), and 80,871 messages from 279 users (Twitter).

The same-context distributions additionally require source posts with at least two distinct human repliers in the held-out test split. Reddit and Twitter/X both contain such contexts (Reddit: 7,932; Twitter/X: 313); Bluesky does not (only one qualifying context) and is therefore excluded from the two distributions that require multiple repliers to the same context (Human–human, same context; AI–AI, same context). Constructing the matched comparisons additionally requires that context to also have ≥ 2 simulated AI users, which only a random subsample of each user's held-out messages was generated for; after this further filtering, 1,371 contexts remain usable on Reddit and 18 on Twitter/X. The third same-context distribution, Intra-AI (same user and context), only requires a single simulated user to have multiple generation candidates for the same context, which is available on all three platforms, including Bluesky.

Embedding procedure. All unique texts appearing in a platform's entries (human ground-truth messages, selected AI responses, all candidate responses, and all training-split human messages) are deduplicated and encoded in a single batch with batch size 512, yielding a shared embedding lookup used for every distribution below.

Distributions. We compute the following distributions. The first five require no shared-context data and are sampled to n observations equal to the number of test entries for that platform; the last two are computed only over contexts with the required number of repliers (Reddit and Twitter/X) or, for Intra-AI (same user and context), over every test entry with at least two generation candidates (all three platforms).

1. **Human-vs-AI**. For each test entry i, we compute $\cos(\mathbf{h}_i, \mathbf{a}_i)$ where $\mathbf{h}_i$ is the embedding of the human ground-truth message and $\mathbf{a}_i$ is the embedding of the selected AI response. This is the primary quantity of interest; the other distributions serve as reference points.

2. **Intra-AI (same user and context)**. For each test entry we draw one random pair from its pool of (up to 20) candidate responses and compute their cosine similarity. This measures how similar two AI responses are when generated from the *same* prompt, model, and simulated user, capturing intra-model consistency. All candidate models are evaluated on the same (context, user) pairs, so this and the two distributions below are computed within a single model at a time and keyed by (model, context, user); pooling across models without this conditioning would let one model dominate the sample.

3. **Intra-human**. We repeatedly draw a user uniformly at random from those with ≥ 2 training messages, then sample two of their messages without replacement and compute their cosine similarity. This reflects topical and stylistic coherence within the writing of a single person, but conflates person and topic (it does not hold context fixed).

4. **Random-human**. We draw two messages uniformly at random from the full training corpus (without user constraints) and compute their cosine similarity. This is the cross-user, cross-context human floor.

5. **Random-AI**. We draw two selected AI responses uniformly at random from different test entries and compute their cosine similarity. This is the cross-context AI floor.

6. **Human–human (same context)**. For every context with at least two human repliers, we sample one pair of distinct users' replies and compute their cosine similarity. Unlike Intra-human, this holds context fixed and varies the author, isolating the effect of shared context from shared authorship. Reddit and Twitter/X only.

7. **AI–AI (same context)**. Two different simulated users' AI replies to the same context, same model. The AI-side counterpart to Human–human (same context). Reddit and Twitter/X only.

All random sampling uses a fixed seed for reproducibility.

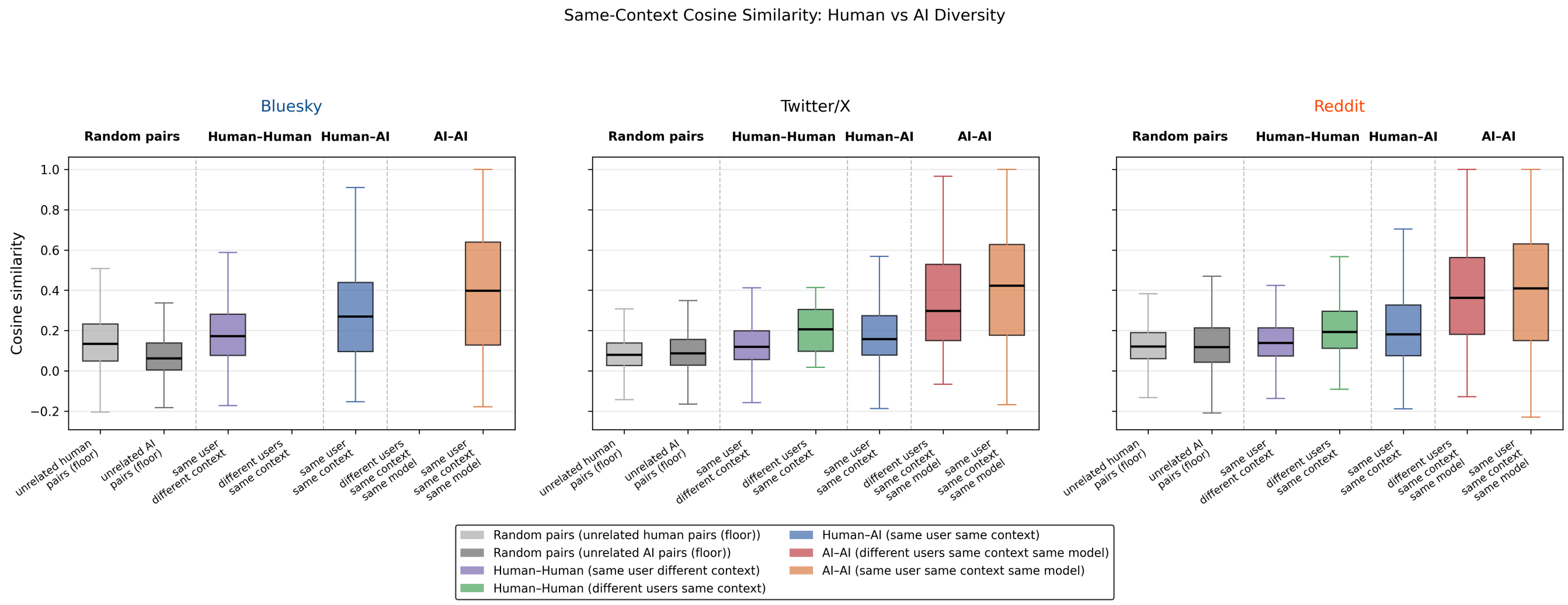

Fig. S6. Cosine similarity distributions under the reference configuration, one panel per platform (order: Bluesky, Twitter/X, Reddit). Boxes are grouped by comparison type: random pairs (floor), human–human (intra-human and, where computable, same context), human-vs-AI, and AI–AI (same context, and intra-AI same user & context). The two same-context human boxes are empty for Bluesky, which lacks contexts with multiple human repliers (Twitter/X same-context boxes: $n = 18$, exploratory).

Results: reference configuration. Figure S6 shows the cosine similarity distributions for each platform under the reference configuration. Note that the two same-context human distributions are based on many fewer observations than the other distributions, since they require a source post with multiple repliers: 1,371 pairs on Reddit, but only 18 on Twitter/X (from 313 qualifying contexts, most of which did not yield a usable pair after filtering). At $n = 18$, the Twitter/X same-context statistics are exploratory rather than confirmatory – we verified directly that a single multi-reply post's phrasing can shift the reported median by more than 10 percentage points – whereas the Reddit same-context statistics ($n = 1{,}371$) are well powered and should be treated as the reliable estimate throughout this section.

Several consistent patterns emerge across platforms.

AI–AI similarity, at same context or same user, is well above the human–human same-context baseline. On Reddit and Twitter/X, both AI–AI (same context) and Intra-AI (same user & context) substantially exceed Human–human (same context): 1.5–1.9× for cross-user AI pairs, and higher still when the same simulated user is resampled. This is calibrated evidence for the homogenization we otherwise describe only qualitatively: different simulated personas (and, even more so, repeated draws from the same persona) converge toward each other in embedding space to a degree that two independent human authors replying to the same post do not.

Human-vs-AI similarity is consistently above the cross-context human floor, but below the same-context human baseline. Median human-vs-AI similarity (0.157–0.269) exceeds both the intra-human baseline (0.119–0.172) and the random-human floor (0.080–0.133) on all three platforms, so AI responses are not merely plausible on-topic text: they are closer to the specific human message they were tasked to mimic than two arbitrary messages by the same author. On Reddit and Twitter/X, however, human-vs-AI similarity (0.181, 0.157) remains below the same-context, different-user human baseline (0.193, 0.206) under the reference configuration – two real humans replying to the same post are, on average, still more similar to each other than an AI reply is to the human it is meant to emulate.

Random-AI and random-human floors are similar to each other and close to zero. Cross-context AI responses (median 0.061–0.118) and cross-user human messages (median 0.080–0.121) are approximately equally similar to one another, and near the lower bound of the similarity scale, confirming that both floors are appropriately uninformative baselines.

Results: calibration depth. We repeat the same-context comparison at three levels of calibration – the reference configuration (Baseline+Persona), the addition of stylistic examples and context retrieval (+Style+Context), and fine-tuning (+Fine-tuning) – to test whether homogenization changes as models are calibrated to individual users. Figure S7 reports median cosine similarity for Reddit and Twitter/X (Bluesky is excluded throughout, for the reason given above).

The AI–AI and intra-AI homogenization gap narrows steadily with calibration depth and is essentially closed by fine-tuning on Reddit, where both converge to the human–human baseline. Under +Style+Context, human-vs-AI similarity on Reddit briefly exceeds the human–human same-context baseline.

9. Feature-level stylistic bias under optimal configurations

Figure S8 mirrors the feature bias analysis of Section 7, but replaces the fixed reference configuration with each model's individually optimal configuration. Each panel shows the distribution corresponding to AI-generated and human-written text for a single feature, platform, and model. Feature columns are ordered by mean Random Forest importance (left = most discriminative) under the best-configuration setting; a feature must enter the globally-derived candidate set—top 10 across all

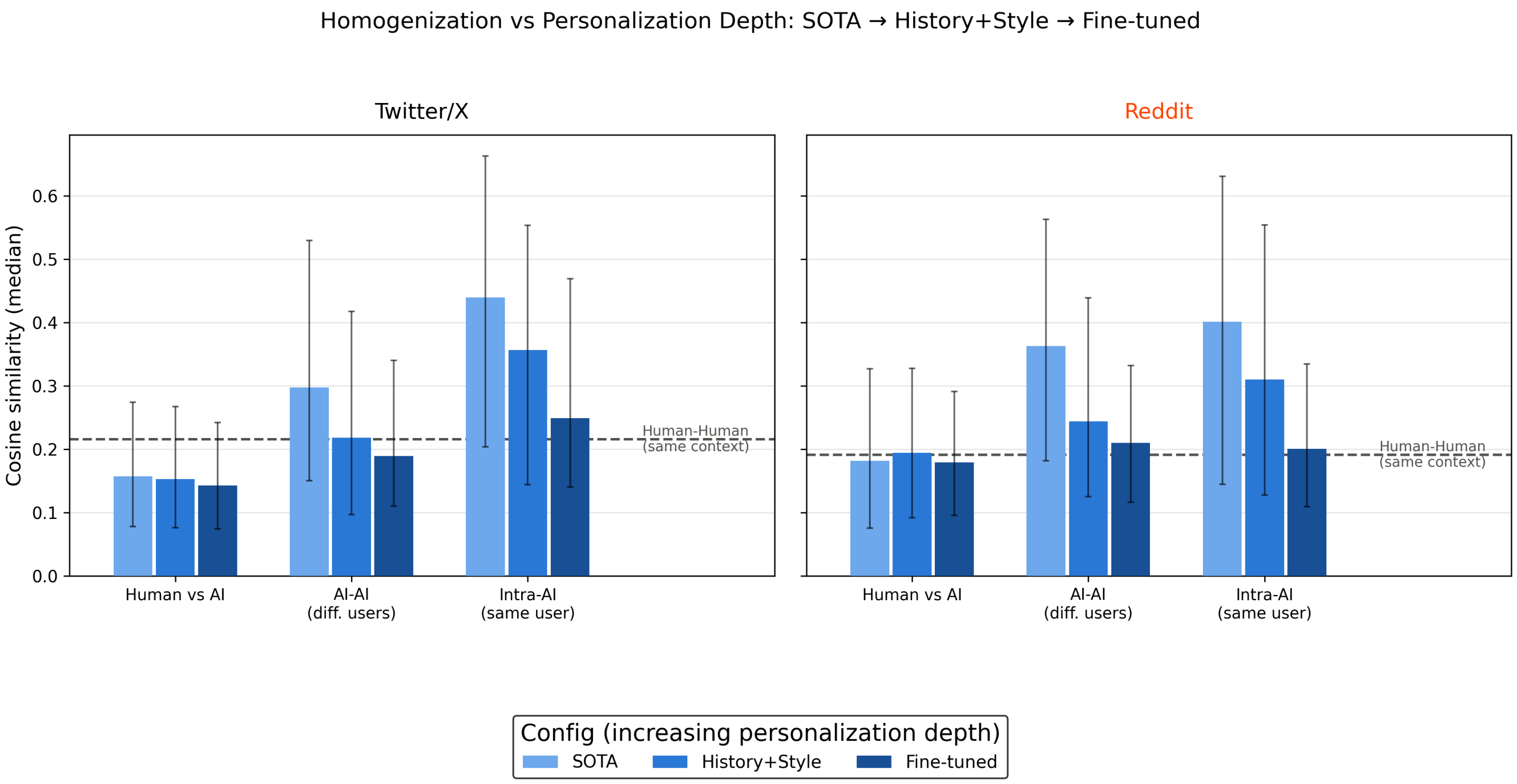

Fig. S7. Same-context cosine similarity distributions across three levels of calibration (Reddit and Twitter/X only). AI–AI and Intra-AI similarity decrease toward the Human–human same-context baseline as calibration deepens; Human–human (same context) does not depend on the AI configuration and is shown as a reference line.

platforms and models plus top 2 per model—to be included, following the same selection rule applied to the explainability heatmaps.

Persistent biases. The dominant pattern is that most stylometric biases documented under the baseline configuration are *not* resolved by configuration optimization. Toxicity scores remain systematically below the human reference on all three platforms: safety-aligned models produce near-zero toxicity regardless of whether style, context, or fine-tuning are added, confirming that this is a model-level property rather than a configuration artefact. Similarly, word count, average word length, perplexity proxy, type-token ratio, and uppercase ratio deviations persist, even though differences are reduced in magnitude (see for example the comparison with S5 on word count on Bluesky).

Changes relative to baseline. Two structural differences distinguish the feature set here from the reference configuration analysis. First, *sentiment* enters as a top discriminative feature on all three platforms, replacing sentence count and (on Bluesky and Twitter) mention count. This shift might indicate that the best configurations include style enrichment for most models, which conditions outputs on the target user's emotional register; the resulting outputs are more tonally varied than baseline responses, making sentiment a newly informative classifier signal. Alternatively, it might also mean that calibration does not manage to mitigate differences in sentiment, while it manages to mitigate them for other features that are becoming less important. Second, *syntactic complexity* appears among the top features on Bluesky and Reddit. Models operating under context-enriched configurations produce longer, more syntactically elaborate outputs, widening the gap relative to the typically sparse and informal human replies on those platforms.

Mention overuse under best configurations. The disappearance of mention count from the top feature set for most platforms indicates a meaningful improvement: under the best configuration, instruction-tuned models that previously hallucinated @mentions at rates an order of magnitude above the human baseline substantially reduce this behaviour. Style and context conditioning appear to anchor the models more strongly to the platform-specific reply register, suppressing the format hallucinations that were prominent under the baseline.

10. Cosine similarity under optimal configurations, by model

Figure S9 decomposes the cosine similarity between AI-generated and human replies across models and response-selection strategies, for each platform separately. Each model group contains four distributions: the reference configuration (BL+PE), the best-configuration random response, the best-configuration cosine-optimal response, and the best-configuration ML-optimal response. The best configuration is defined per model and platform as the setting maximising BERT-classifier accuracy on the random response type.

Effect of configuration on cosine similarity. Moving from the baseline to the best random configuration does not produce any clear improvement in cosine similarity for the majority of models and platforms. Instead, switching from random response

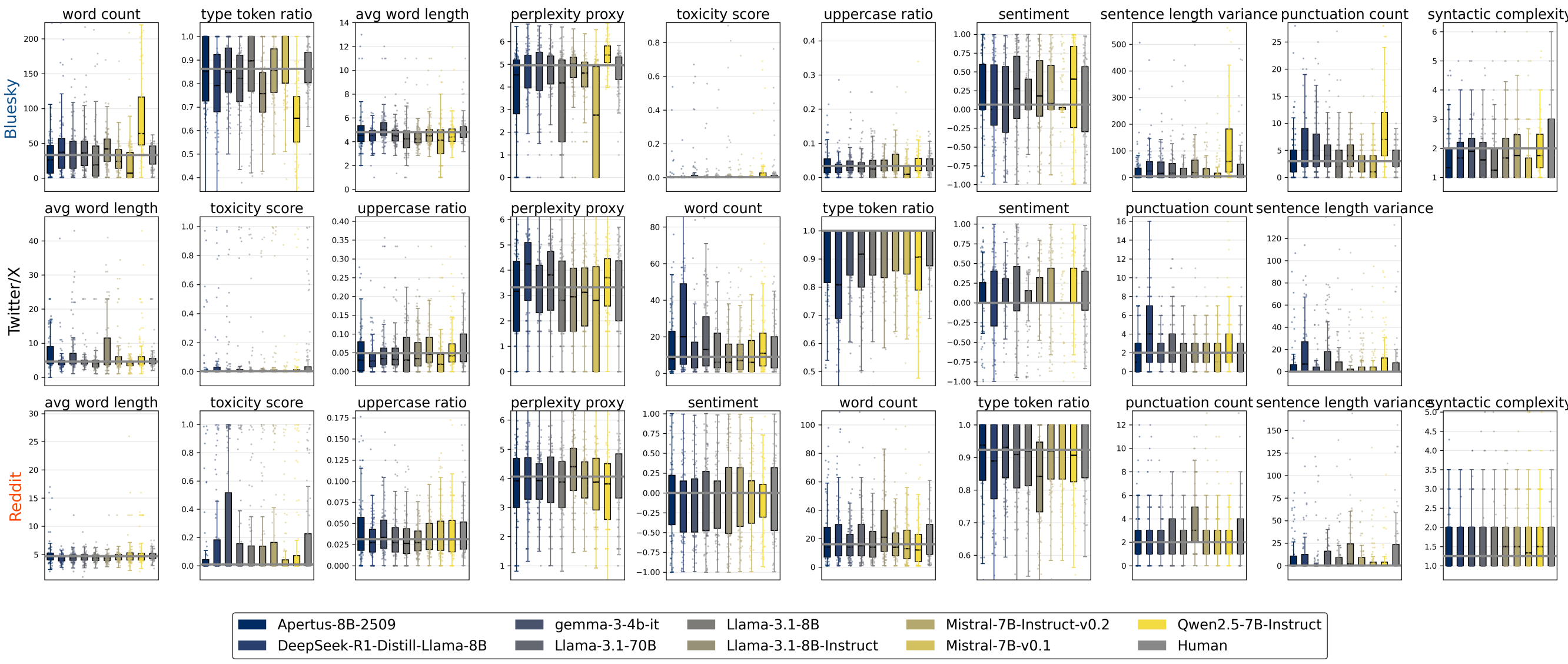

Fig. S8. Stylometric feature bias for each model under its individually optimal configuration, per platform (rows) and feature (columns). Each boxplot shows the distribution across all responses generated by that model and in the human sample (in grey); the dashed line indicates the human median. Features are ordered (platform-wise) left-to-right by mean Random Forest importance under the best-configuration setting. Twitter contains nine feature columns rather than ten because the globally-derived candidate set yields only nine eligible features for that platform (selection was based on top 10 most important features, provided that they are relevant for at least 2 models). Box colours correspond to models (see legend); jittered strip overlays show individual response-level values (up to 80 points per model, sampled uniformly). y-axes are clipped to the 2nd–98th percentile of the combined distribution to suppress extreme outliers.

selection (from best configuration) to cosine-optimal provides a clear improvement. The cosine-optimal selector outperforms random selection more uniformly across models and platforms, as it directly optimises the metric being plotted; the ML-optimal selector shows comparatively smaller gains on average, suggesting that the ML ranking criterion (classifier confidence) does not systematically identify responses that are stylistically closer to the human reference. Across all three selection strategies the between-model variance remains relatively small (with only Mistral-7B-v0.1 and Llama-3.1-8B performing significantly worse than the other models on Bluesky and Reddit data): the ordering of models by cosine similarity is approximately preserved regardless of which selector is used, indicating that model-level stylistic capacity is the dominant factor and that response selection provides a second-order correction on top of it.

11. Fine-Tuning Hyperparameter Ablation

To investigate whether the variation in fine-tuning detectability results is attributable to hyperparameter choice, we conducted an ablation study varying the LoRA configuration and learning rate across three alternatives designed to mitigate potential overfitting: (i) a low-rank adapter (r=4, α=8), (ii) a conservative learning rate (r=8, lr=5×10^{-5}), and (iii) a higher dropout coefficient (r=8, dropout=0.2). We focused on the two models where fine-tuning had the largest impact on detectability: Llama-3.1-8B and Mistral-7B-v0.1 on Twitter.

Results are shown in Figures S10a–S10b. Hyperparameter choice has negligible effect: for Mistral-7B-v0.1, classifier accuracy is essentially unchanged across all variants (0.972–0.973), and for Llama-3.1-8B the best alternative yields only a modest reduction relative to the default (-0.014), leaving accuracy at $\approx$0.95. Standard deviations over three repeated runs are very small ($\leq$0.003), confirming that these differences are reliable but minor. Notably, the best no-fine-tuning configuration achieves considerably *lower* classifier accuracy than any fine-tuned variant (0.827 vs. $\approx$0.955 for Llama-3.1-8B; 0.910 vs. $\approx$0.972 for Mistral-7B-v0.1), indicating that fine-tuning makes generated text *more* detectable, not less.

Analysis of stylistic feature importances clarifies why (Figure S10b). Fine-tuning on a small, user-specific dataset does not bring AI text closer to human text on the features the classifier relies on most; instead, it widens several key gaps. Uppercase ratio is the dominant signal across all configurations and both models, but is far more pronounced in fine-tuned variants. Emoji count shows a notably elevated importance for Mistral-7B-v0.1 after fine-tuning, suggesting that the model amplifies idiosyncratic stylistic patterns rather than learning to suppress them.

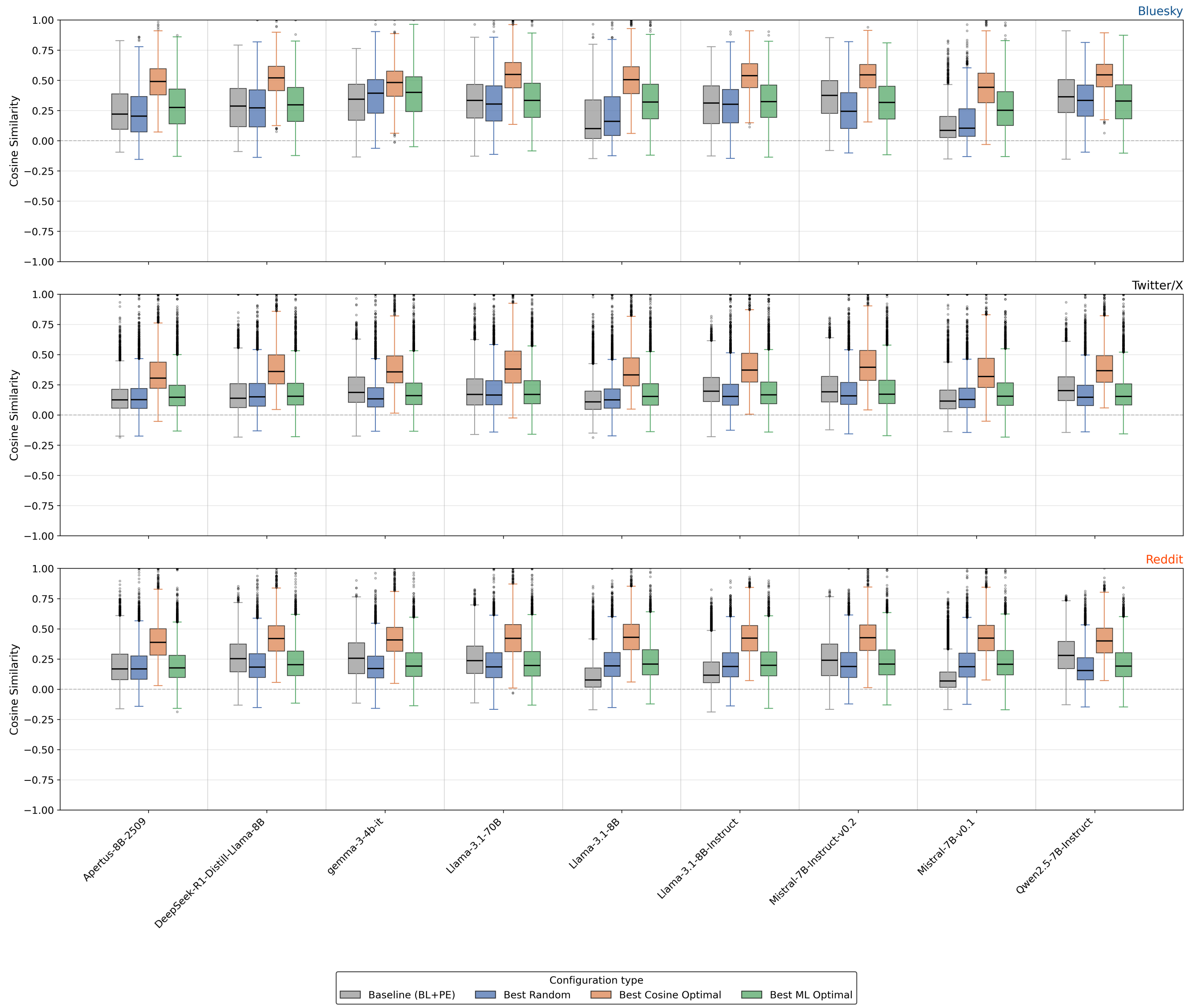

Fig. S9. Cosine similarity between AI-generated and human replies, broken down by model (groups on the x-axis) and response-selection strategy (box colour), for each platform (rows). Gray: baseline/reference configuration (BL+PE); blue: best configuration with random response selection; orange: best configuration with cosine-optimal selection; green: best configuration with ML-optimal selection. The best configuration is chosen per model and platform by maximising random-type BERT classifier accuracy. Model names are coloured by their group affiliation. Boxes span the interquartile range; whiskers extend to 1.5×IQR; the horizontal line inside each box marks the median.

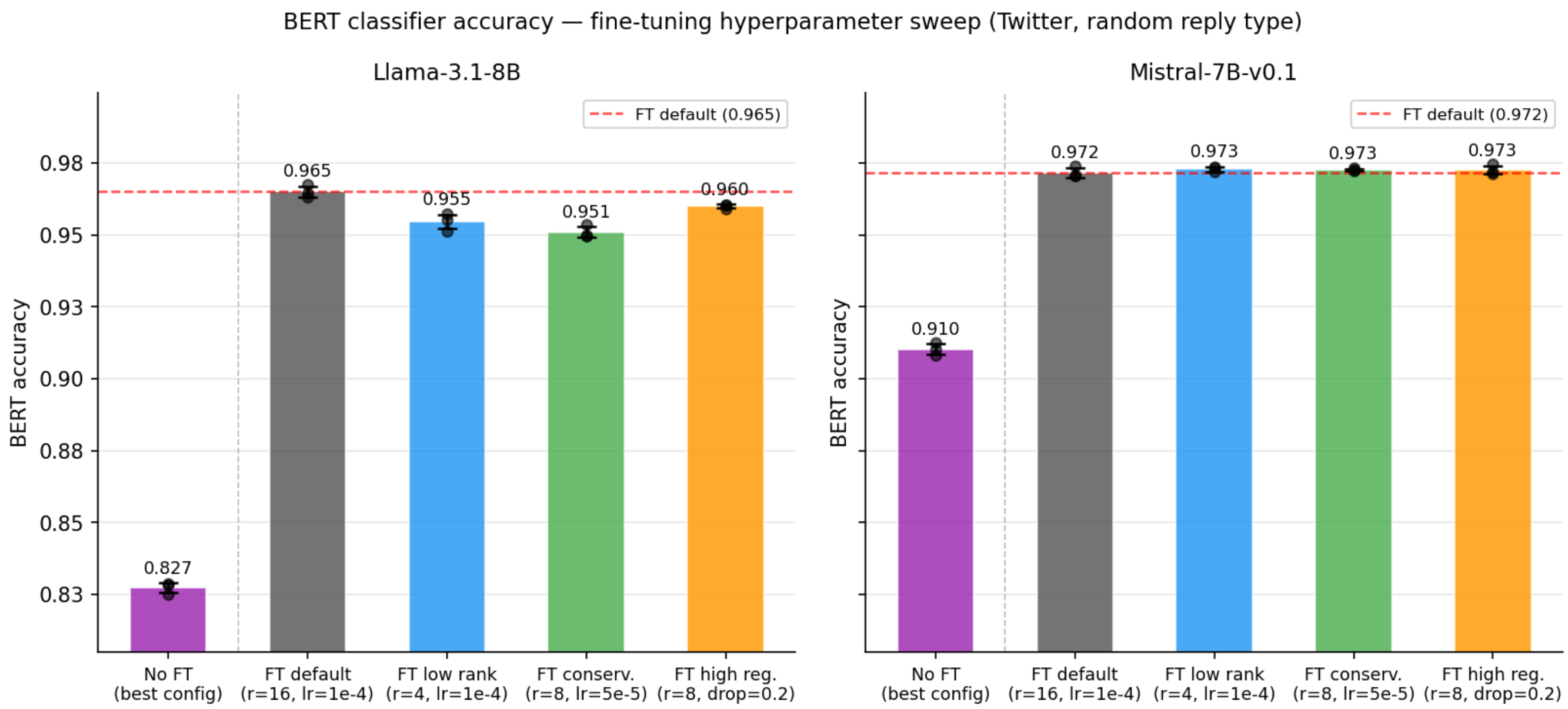

(a) BERT classifier accuracy on Twitter (random reply type; lower is better), comparing the best no-fine-tuning configuration (purple) against fine-tuned variants differing in LoRA rank, learning rate, and dropout. The dashed red line marks the default fine-tuning accuracy for each model.

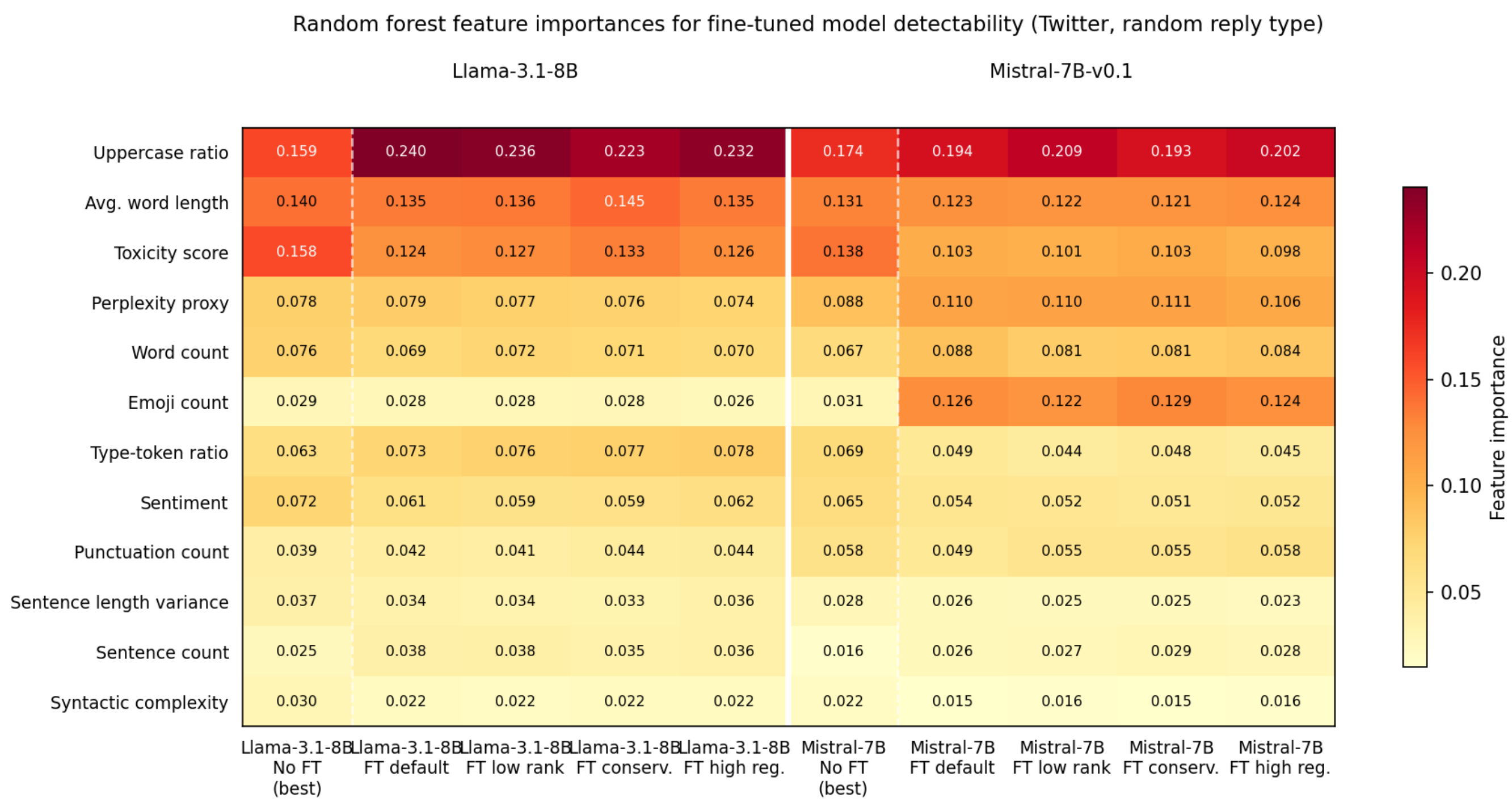

(b) Random forest feature importances for distinguishing AI-generated from human text (Twitter, random reply type). The dashed vertical line separates the best no-fine-tuning configuration from fine-tuned variants.

Fig. S10. Fine-tuning hyperparameter ablation on Twitter (random reply type).

12. Decoding Hyperparameter Ablation

The reference decoding configuration used throughout this work (T=0.8, top-p=0.9, top-k=50) combines three sampling constraints that each independently reduce the entropy of the output distribution. These are, individually, widely used defaults for open-ended sampling rather than values tuned for any particular outcome: top-p=0.9 is the representative setting from the nucleus-sampling paper that introduced it (1), top-k=50 is the default value in HuggingFace's `transformers` generation configuration, and T=0.8 is a commonly used moderate-temperature setting in practical open-ended generation. Combining all three, however, compounds their entropy-reducing effect beyond what any single one would produce alone. To characterize the consequences of this choice directly, we conducted a targeted ablation rather than re-running the full experimental matrix under a different fixed setting, which would simply substitute one arbitrary choice for another.

Ablation design. On Bluesky (59 users), we generated additional response sets under a more permissive decoding configuration, for two models spanning different baseline detectability levels. "Baseline" below is the reference configuration (Baseline+Persona, no fine-tuning, style conditioning, or context retrieval) used throughout the main text and specifically for the accuracies reported in Figure 1.

Table S2. Decoding configurations used in the ablation.

Configuration	Model	T	top-p	top-k
Baseline (reference config., Fig. 1)	Llama-3.1-8B	0.8	0.9	50
Looser	Llama-3.1-8B	1.0	0.95	0 (disabled)
Baseline (reference config., Fig. 1)	Mistral-7B-Instruct-v0.2	0.8	0.9	50
Looser	Mistral-7B-Instruct-v0.2	1.0	0.95	0 (disabled)

Detectability. Table S3 reports BERT classification accuracy (lower indicates harder to detect), separated by which post-generation selection method is being validated. Looser decoding reduces detectability specifically on the two selection pathways reported in the main text (cosine-optimal and ML-optimal), most consistently for ML-optimal selection (a 3.2–3.7 percentage-point drop for both models); the improvement on cosine-optimal selection is more modest (0.5–1.3pp). The one reversal is the unfiltered random pick, which becomes *more* detectable for Llama-3.1-8B as decoding loosens (+2.5pp) – consistent with looser decoding widening the spread of candidates (i.e., reducing homogenization) rather than uniformly improving the quality of any single draw.

Table S3. BERT classification accuracy under each decoding configuration.

Model	Selection method	Baseline	Looser
Llama-3.1-8B	Cosine-optimal	70.8%	69.5%
Llama-3.1-8B	ML-optimal	72.0%	**68.9%**
Llama-3.1-8B	Random (unfiltered)	82.0%	84.5%
Mistral-7B-Instruct-v0.2	Cosine-optimal	96.0%	95.5%
Mistral-7B-Instruct-v0.2	ML-optimal	92.6%	**88.9%**
Mistral-7B-Instruct-v0.2	Random (unfiltered)	96.8%	96.8%

The cost: semantic fidelity. This detectability reduction is not free. Median cosine similarity to the human ground truth (Llama-3.1-8B) decreases as decoding loosens, for every reading we checked: the random pick (0.160 $\rightarrow$ 0.133, baseline/looser), the ML-selected reply (0.321 $\rightarrow$ 0.293), and even the cosine-optimal reply itself (0.505 $\rightarrow$ 0.492). For Mistral-7B-Instruct-v0.2 all three readings are essentially flat rather than improving (Figure S11). In other words, the specific replies our pipeline selects and validates become *less*, not more, semantically related to what the target human actually wrote as decoding is loosened.

Interpretation. Reduced detectability under looser decoding is attributable to a shift in surface-level/stylistic properties (the features our classifier and Random Forest analysis are sensitive to), not to generations becoming more accurate proxies for the target human's response. This is a trade-off, not a free improvement: our reference hyperparameters were not artificially inflating detectability at no cost, and simply increasing sampling entropy is not a way to make LLM-generated text "pass" as human for free. It reproduces, within decoding alone, the same stylistic-realism-vs-semantic-fidelity tension that is a central finding of the main text, and it is discussed further in the Discussion section.

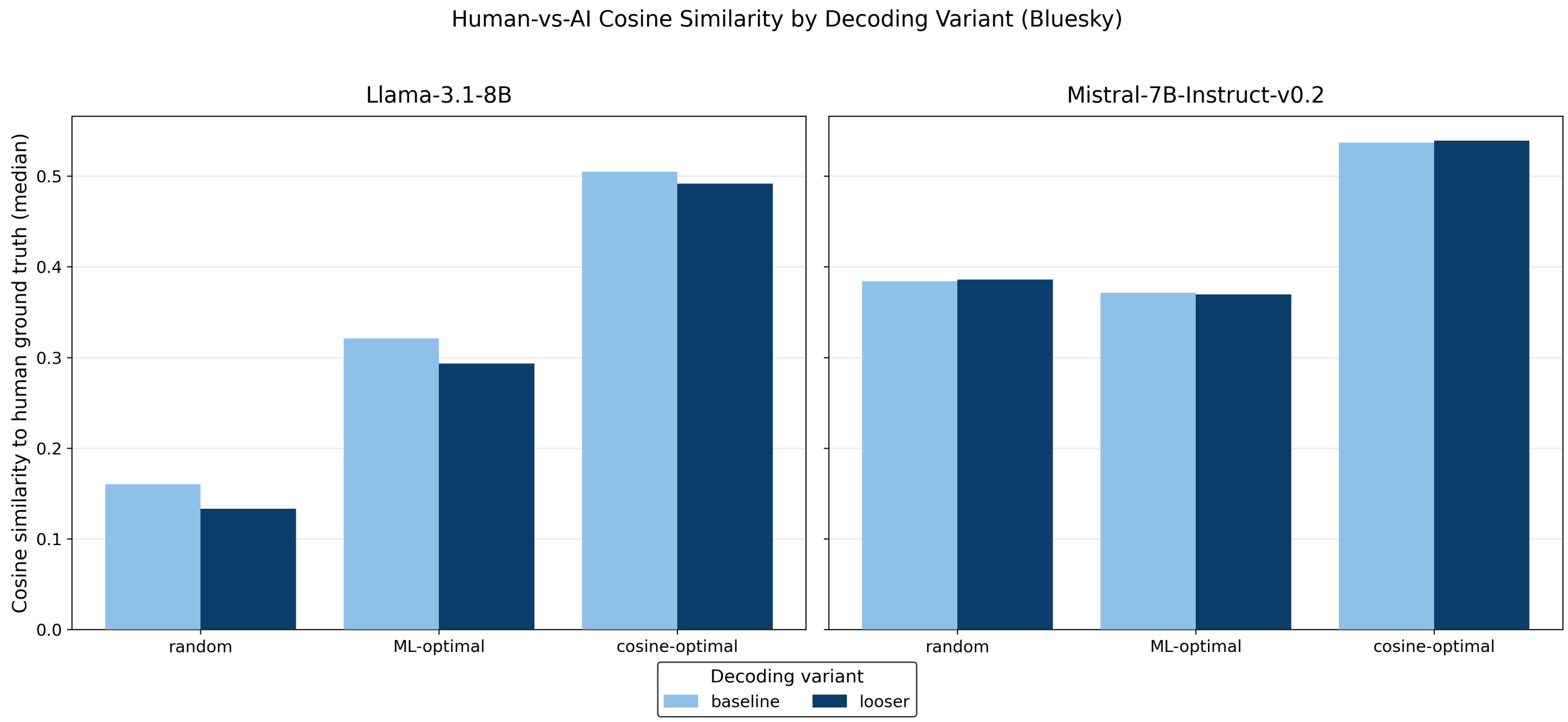

Fig. S11. Median cosine similarity to the human ground-truth reply, Llama-3.1-8B and Mistral-7B-Instruct-v0.2 on Bluesky, across the two decoding configurations in Table S2 and the three reply-selection methods (random, ML-optimal, cosine-optimal). Semantic fidelity decreases or stays flat as decoding loosens; it never improves.

13. BERT Classifier False Negative and False Positive Rates

Because the validation set is exactly balanced (one AI-generated reply paired with the corresponding human ground-truth reply per source message), overall accuracy could conceal an asymmetry between class-specific error rates. We report false-negative and false-positive rates for every model, platform, and configuration, alongside the previously reported accuracy.

A false negative (FN) is an AI-generated reply misclassified as human (detection failure); a false positive (FP) is a genuine human reply misclassified as AI-generated (a wrongly flagged human). For a balanced set, accuracy $= 1 - (\text{FN} + \text{FP})/2$, so FN and FP rates are jointly redundant with accuracy but individually reveal whether errors are concentrated in one direction. Rates are computed from the confusion matrix averaged across the three random seeds used throughout this work (Methods), consistent with all other reported accuracies.

Conditions. We report four conditions per model and platform: the reference configuration (Baseline+Persona); the best-performing calibrated configuration; and that same best configuration combined with each post-generation selection strategy (Cosine-optimal, ML-optimal).

Results. Figure S12 plots FN rate against FP rate for every (model, platform, condition) combination (108 points total), with marker size proportional to accuracy and the diagonal marking FN=FP. In 82 of 108 combinations (76%), FP exceeds FN: the classifier is more prone to wrongly flagging a genuine human reply as AI-generated than to missing an actual AI-generated reply. This holds on average within every platform and condition (mean FN/FP: Reference 3.7%/5.4%; Best config 12.1%/17.0%; Best config+Cosine-optimal 13.9%/20.0%; Best config+ML-optimal 18.5%/24.3%), and is most pronounced where detectability is already high. It weakens or reverses for the non-instruction-tuned models that separately achieve the lowest overall detectability (Llama-3.1-8B, Llama-3.1-70B, Apertus-8B-2509, and Gemma-3-4B on Bluesky/Twitter), where FN and FP rates converge as classifier accuracy approaches chance.

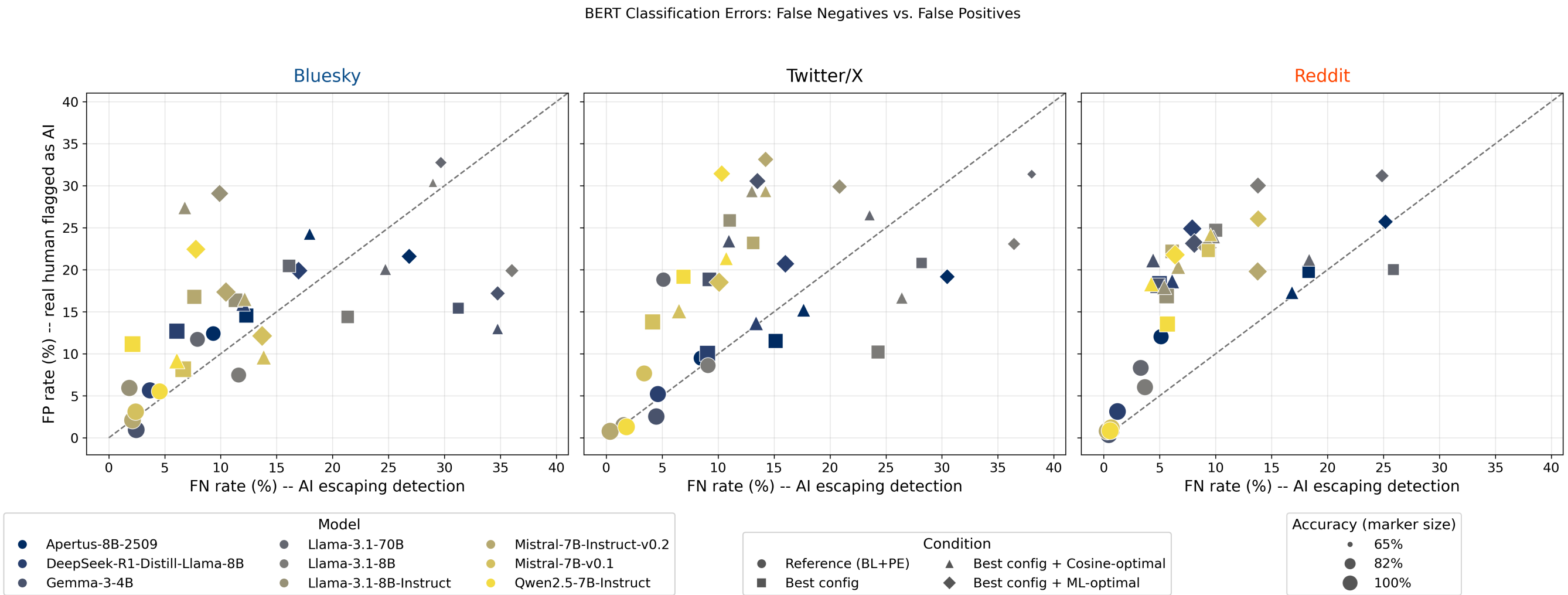

Fig. S12. BERT classifier false-negative rate (AI text escaping detection) vs. false-positive rate (human text wrongly flagged), for every model, platform, and configuration. Marker size is proportional to classification accuracy; the dashed line marks FN=FP.

References

1. A Holtzman, J Buys, L Du, M Forbes, Y Choi, The curious case of neural text degeneration in *International Conference on Learning Representations*. (2020).
